# Interpersonal Problems and Their Mental Health Correlates: A Meta‐Analytic Review

**DOI:** 10.1002/jclp.70022

**Published:** 2025-08-04

**Authors:** Flavio Iovoli, Julian A. Rubel, Tobias Steinbrenner, Ruben Lauterbach

**Affiliations:** ^1^ Department of Psychology Osnabrueck University Osnabrueck Germany; ^2^ Department of Medicine Justus‐Liebig‐University Giessen Giessen Germany

**Keywords:** anxiety, depression, interpersonal problems, mental health, meta‐analysis, psychological distress

## Abstract

Interpersonal relationships have long been considered fundamental to understanding mental health and psychological functioning. Among various aspects of these relationships, interpersonal problems seem particularly significant in relation to mental health. This meta‐analytic review aims to summarize the associations between interpersonal problems and multiple domains of mental health based on naturalistic, observational data from both clinical and nonclinical populations. Focusing on studies using the Inventory of Interpersonal Problems (IIP), we examined the cross‐sectional associations between interpersonal problems and general psychological distress, depressive symptoms, symptoms of anxiety, positive and negative emotions, well‐being, and perceived stress. A three‐level meta‐analytic model was employed to account for the nested data structure, and moderator analyses examined differences between clinical and nonclinical samples. Across all domains, 120 effect sizes from 66 studies were included. Significant positive correlations emerged between interpersonal problems and general psychological distress (*r* = 0.565, *p* < 0.001), depression (*r* = 0.473, *p* < 0.001), anxiety (*r* = 0.454, *p* < 0.001), and negative emotions (*r* = 0.486, *p* < 0.001). In contrast, significant negative correlations were found with positive emotions (*r* = −0.224, *p* = 0.010) and well‐being (*r* = −0.372, *p* = 0.004). There were too few studies available to meta‐analyze perceived stress. Clinical status did not significantly moderate these associations, suggesting that the observed associations are relatively consistent across populations. These findings underscore the relevance of interpersonal problems, as measured by the IIP, for understanding mental health and provide evidence‐based estimates of these associations.

## 1 Introduction

Interpersonal relationships are crucial to human development and psychological functioning (Argyle 2017; Neuberg et al. 2010; Sullivan 1953). They shape how individuals relate to others and perceive themselves (Pincus and Ansell 2003). The significance of these relationships has been a central focus in interpersonal theory pioneered by Sullivan (1953) and Leary (1957). They offer an exploration of how interpersonal dynamics contribute to mental health and the development of maladaptive relational patterns (Horowitz 1996; Horowitz and Vitkus 1986). For instance, Sullivan (1953) proposes three potential outcomes of interpersonal situations that are central to understanding how relationships impact mental health (Pincus and Ansell 2003). He argues that interpersonal situations are patterns (or alternations) of relating between the self and others, which is associated with varying levels of anxiety and security (Evans 2020). The first outcome occurs when interpersonal needs between individuals are fulfilled, leading to mutual satisfaction which creates a feeling of security. These situations are likely to recur. The second outcome arises when the needs or behaviors between individuals are not immediately compatible, which causes tension. The tension associated with this unresolved state can lead to varying levels of anxiety (Pincus and Ansell 2003). If the parties involved can negotiate and adapt their behavior, the interaction might eventually lead to a resolution (Kiesler 1996). The third and most detrimental outcome is when interpersonal needs are neither compatible nor resolved (Pincus and Ansell 2003). The tension escalates as no resolution was found, and it results in the disintegration of the relationship (Horowitz 1996). Sullivan (1953) emphasized that when interpersonal situations reach this point, anxiety can become overwhelming, and lead to lasting psychological impacts and general psychological distress. In turn, this can reinforce negative self‐perceptions and exacerbate mental health issues, such as depression or anxiety (Evans 2020; Pincus and Ansell 2003).

Building on these concepts, Leary (1957) expanded on the interpersonal theory with the concept of complementarity, which describes how interactions match and influence each other along two primary dimensions: affiliation (also referred to as communion) and dominance (or agency; Gurtman 2020). Affiliation refers to the spectrum of behavior from hostile to friendly, whereas dominance ranges from dominant to submissive (Gurtman 2009). According to Leary (1957) model, interpersonal behaviors are typically complementary. In other words, one individual's behavior invites a specific (complementary) response from the other, also referred to as “interpersonal pull” (Horowitz et al. 1997; Kiesler 1983; Sadler and Woody 2003). While on the dominance dimension a certain behavior evokes the respective contrary behavior in the other person, behaviors on the affiliation domain evoke similar behaviors (Wiggins 1991).

Continuing from the concept of complementary interpersonal interactions, these patterns can have a profound impact on one's mental well‐being. To give an example, Horowitz (1996) outlines how a hostile submissive interpersonal style elicits a hostile dominant response, and that this response might be perceived as harsh and domineering. In turn, the response invites again the original hostile submissive interpersonal style, and traps the individual in a vicious cycle (Horowitz 1996). A hostile submissive interpersonal style is thought to be highly stable, and causes the individual to experience the interactions as stressful and dissatisfying (Sadler and Woody 2020). Consequently, these interactional patterns negatively reflect on the self, where the individuals find themselves passive, helpless, with self‐accusive and punishing thoughts, and anticipate future interactions to be similar in nature (Horowitz 1996; Horowitz et al. 1997). Therefore, the experience of interpersonal problems is likely to be associated with psychopathological symptoms and one's general well‐being (Sadler and Woody 2020; Segrin and Taylor 2007).

To systematically assess these kinds of interpersonal problems, the *Inventory of Interpersonal Problems* (IIP) was developed (Horowitz et al. 1988). The items of the IIP were constructed based on interviews with outpatients who reported their issues seeking treatment. Judges then rated if the problem was interpersonal related or not (Horowitz 1979). In its original form, the IIP lists 127 statements that assess the individuals' typical behavioral excesses (doing something too much) and inhibitions (something is hard for them; Horowitz et al. 1997). The IIP allows the combination of the two dimensions, which results in a circumplex model with eight octants: domineering, intrusive, overly nurturant, easily exploitable, nonassertive, socially avoidant, cold, and vindictive (Alden et al. 1990; Horowitz et al. 1997; Wiggins 1991). In addition, the global score is commonly interpreted as the severity of interpersonal problems. In this context, interpersonal problems, as assessed with the IIP, refer to the enduring difficulties an individual typically experiences in relation to others and perceives as distressing (Horowitz et al. 1988). The severity of these problems has been shown to represent a relatively stable personality trait (Quilty et al. 2013; Wright et al. 2013). To date, the IIP remains the most commonly used instrument to operationalize interpersonal problems. It is particularly prevalent in meta‐analytic research, where it often serves as the primary indicator of interpersonal problems (e.g., Gómez Penedo et al. 2025; Gómez Penedo and Flückiger 2023; Iovoli et al. 2024; Liebherz and Rabung 2014; McFarquhar et al. 2018). In addition, the instrument is available in several versions, varying in length, all of which have demonstrated good reliability (Horowitz et al. 2000; Lutz et al. 2006; Soldz et al. 1995).

Previously, it has been shown that interpersonal problems are frequent complaints treatment‐seeking individuals report (e.g., Grosse and Grawe 2002; Horowitz 1979) and are prominently featured in several theories of depression (for overview, see McFarquhar et al. 2018). For example, Lewinsohn. (1974) behavioral theory suggests that depressive symptoms persist due to low positive reinforcement in social interactions, while others highlight the role of negative interpersonal feedback (e.g., Joiner 2000; Swann et al. 1990). Consistent with these models, greater interpersonal problems, as measured with the IIP, have been consistently associated with higher levels of depression (e.g., Connolly Gibbons et al. 2003; Grosse Holtforth et al. 2014), even in nonclinical samples (e.g., Huprich et al. 2016). Similar associations have been found for anxiety symptoms, where interpersonal problems may exacerbate anxious patterns such as hypervigilance to social threat or withdrawal (Erickson et al. 2023; Kim and Bae 2022; Malivoire and Koerner 2022; Newman et al. 2008, 2013; Shin and Newman 2019).

Beyond disorder‐specific symptoms, there is growing recognition that interpersonal problems are also linked to broader indicators of mental health and psychological distress (Segrin 1998; Skowron et al. 2009). Psychological distress, in this context, refers to a general state of emotional suffering that encompasses a wide range of psychological and somatic complaints (Drapeau et al. 2011). For example, the Symptom Checklist‐90 (Derogatis 1977; Franke 2002), a commonly used instrument in this domain, includes dimensions such as depression, anxiety, somatization, interpersonal sensitivity, and psychoticism. Because interpersonal relationships are central to everyday life, it is plausible that interpersonal problems are not confined to specific diagnoses but are relevant across a broad range of mental health difficulties. Accordingly, they are increasingly considered a transdiagnostic factor (e.g., Girard et al. 2017; McEvoy et al. 2013; Wendt et al. 2019). This is reflected in associations with quality of life, positive and negative emotions, perceived stress, and general well‐being, which are important components of mental health that extend beyond formal psychiatric diagnoses (e.g., Rakhimov et al. 2023; Ringwald et al. 2024).

Given this broader relevance, our study aims to summarize the associations between interpersonal problems and various domains of mental health. Mental health is increasingly understood as a multidimensional construct that includes both psychological distress and mental well‐being (World Health Organization 2022). From a transdiagnostic perspective, it is therefore essential to consider a range of mental health states beyond disorder‐specific symptoms. Our meta‐analysis seeks to extend this understanding by examining these associations in a naturalistic, observational context; that is, in clinical and nonclinical populations without the influence of controlled experimental conditions or interventions. Accordingly, our guiding research question is: What are the associations between interpersonal problems, as measured by the Inventory of Interpersonal Problems (IIP), and various aspects of mental health, including general psychological distress, depression, anxiety, positive and negative emotions, well‐being, and perceived stress?

To enhance the applicability of our findings, we will conduct a moderator analysis to explore how the association between interpersonal problems and mental health varies between clinical and nonclinical populations. Based on the theoretical framework outlined earlier, which suggests that interpersonal problems are broadly relevant across the spectrum of mental health, we anticipate that the relationship between interpersonal problems and mental health will be consistent across both clinical and nonclinical populations.

## 2 Methods

The current meta‐analysis was preregistered in the International Prospective Register of Systematic Reviews (PROSPERO, CRD42024563448). We share the search strategy, the search results (.*ris* files), the included studies with the extracted information highlighted, and the data set on Open Science Framework (OSF, https://osf.io/tge69/).

### 2.1 Literature Search and Procedure

A systematic literature search was conducted across APA PsycInfo, Web of Science, and PubMed for the relevant literature that included interpersonal problems and mental health. Following an update of our initial search conducted on July 1, 2024, we included studies published in English or German through early September 2024. Inclusion criteria were studies that (a) examined the relationship between interpersonal problems and mental health, (b) provided sufficient information to quantify the relationship, (c) assessed interpersonal problems with a version of the *Inventory of Interpersonal Problems* (IIP), (d) examined at least one mental health domain including general psychological distress, depression, anxiety, mood, affect, life satisfaction, quality of life, or well‐being (mental health *states*), (e) assessed all constructs via self‐reported, (f) were observational in nature or interventional only if the relationship was assessed before any intervention, ensuring we captured only naturally occurring relationships between the two constructs, (g) included exclusively adults (18 years old or older), and (h) where a full‐text was available. Exclusion criteria were (1) systematic reviews, meta‐analyses, case studies, study protocols, book chapters, qualitative studies, (2) studies in population with medical diseases, and (3) studies with less than 50 participants (Lin 2018).

The following search terms were entered into the databases with the additional language filter set to English and German only: (((interpersonal relation*) OR (interpersonal problem*) OR (interpersonal difficult*) OR (interpersonal dysfunction) OR (interpersonal conflict) OR (interpersonal distress) OR (relational difficult*) OR (relational defici*) OR (relational distress) OR (relationship problem*) OR (relationship difficult*) OR (relationship defici*) OR (relationship distress) OR (interactional problem*) OR (interactional difficult*) OR (interactional defici*) OR (interactional distress)) AND ((Inventory of Interpersonal Problems) OR (IIP)) AND ((mental health) OR (mental problems) OR (mental disorder) OR (mental illness) OR (psychological health) OR (psychological distress) OR (psychological problems) OR (psychopathology) OR (well‐being) OR (quality of life) OR (life satisfaction) OR (affect*) OR (depress*) OR (mood) OR (stress) OR (anxi*))) for PubMed (*s* = 1412) and APA PsycInfo (*s* = 4474). The search term for Web of Science was ((ALL = (interpersonal relation* OR interpersonal problem* OR interpersonal difficult* OR interpersonal dysfunction OR interpersonal conflict OR interpersonal distress OR relational difficult* OR relational defici* OR relational distress OR relationship problem* OR relationship difficult* OR relationship defici* OR relationship distress OR interactional problem* OR interactional difficult* interactional defici* OR interactional distress)) AND ALL = (Inventory of Interpersonal Problems OR IIP)) AND ALL = (mental health OR mental problems OR mental disorder OR mental illness OR psychological health OR psychological distress OR psychological problems OR psychopathology OR well‐being OR quality of life OR life satisfaction OR affect* OR depress* OR mood OR stress OR anxi*). This resulted in *s* = 1383 search results. Overall, the systematic literature search resulted in 7269 studies.

In the next step, the three *ris*‐files were uploaded into Citavi (version 6.14). The software automatically detected *s* = 1467 duplicates, which were removed. Therefore, *s* = 5802 entered into the abstract screening. The studies were screened by the first and last author. The abstract screening led to *s* = 4977 being excluded. The remaining *s* = 825 were evaluated for eligibility. The number of studies in the full‐text screening is relatively high because the authors were careful to exclude studies earlier in the process, knowing that the relationship between interpersonal problems and mental health is often only mentioned as a secondary finding (e.g., in the correlation matrix or Supporting Information S1).

Data extraction was guided by a predetermined codebook and decision tree. The extracted data included: authors (with a distinguishing letter added if there were multiple entries), country of origin, sample size, mean age, standard deviation of age, percentage of female participants, population type, clinical status of the population (coded as clinical or nonclinical, if clinical the main diagnoses was coded), version of the Inventory of Interpersonal Problems used, the mental health measure, the specific mental health domain represented by that measure (as described by the authors) and the cross‐sectional correlation coefficients. The mental health measure and its domain were coded using a bottom‐up approach, meaning that instead of using predefined categories, we coded the mental health domains according to the specific descriptions provided by the authors in each study. This way, the domains were categorized based directly on the study descriptions rather than applying a predefined framework.

**Figure 1:**
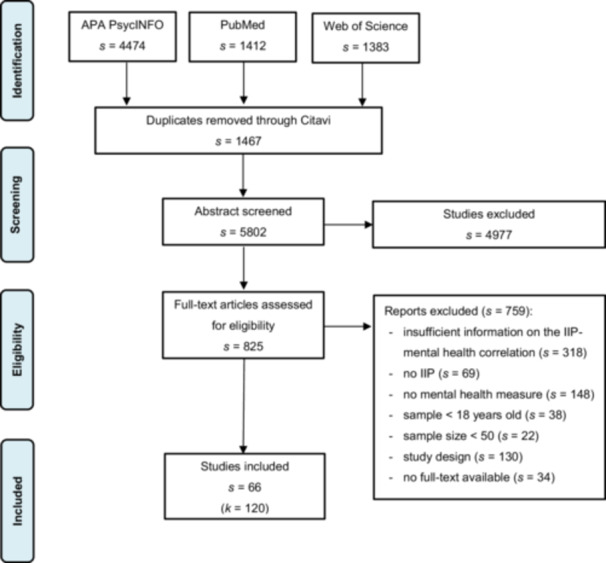
Flow chart. *Note:* IIP = Inventory of Interpersonal Problems. Coding was based on a decision tree. Studies not meeting the required inclusion criteria were excluded. Some exclusion reasons may contain studies that did not meet several criteria but were categorized under one exclusion reason due to the order in the decision tree.

The coding procedure was designed and carried out by the first and last author, which was monitored in frequent consensus meetings. Corresponding authors were contacted to request full‐texts or to clarify potential overlapping samples in multiple studies. If studies used data from the same site (e.g., the same outpatient clinic), the effect size was only included under the following conditions: (a) the study focused on a different mental health domain, or (b) the assessment periods did not overlap. One journal was contacted due to missing material. Both coders independently coded 100 overlapping studies to evaluate inter‐rater reliability. Disagreements were discussed until consensus. The coders achieved good interrater reliability (Cohen's *κ* = 0.83). The extracted data was double‐checked by both coders to ensure accuracy. This process resulted in the inclusion of a total of *s* = 66 studies, incorporating *k* = 120 correlation coefficients into the analysis. The included studies were categorized by mental health domain, resulting in *k* = 30 for general psychological distress, *k* = 39 for depressive symptoms, *k* = 26 for symptoms of anxiety, *k* = 9 for positive emotions, *k* = 7 for negative emotions, *k* = 7 for well‐being, and *k* = 2 for stress. The flow chart is represented in Figure 1.

### 2.2 Statistical Analysis

All analyses were performed using *R* (version 4.3.2, R Core Team 2018). Pearson's correlation coefficients were the effect size of interest. When studies explicitly reported correlations between interpersonal problems and a mental health domain, these were directly included in the data set. For other types of effect sizes, conversions were applied using the standard formulas from Borenstein et al. (2009), utilizing the *R* package *MAc* (version 1.1.1; Del Re and Hoyt 2018). In cases where no correlation coefficients were reported but data was publicly available, we calculated the correlations based on the provided data (*s* = 1). In these cases, we added the R code and output to the study which we made available on OSF. The construct of interpersonal problems was defined as the total (summary or mean) score of the IIP or the elevation score if authors used the structural summary method as the indicator for distress (SSM; Gurtman 1992a). If studies reported the correlations between the eight octant scales and the mental health measure, we computed the elevation score using the formula provided in Zimmermann and Wright (2017; in their Appendix, formula A1). This procedure was only done if subscale scores were not ipsatized, which removes the general distress (or elevation) information (Becker and Mohr 2005; Grosse Holtforth et al. 2006; Thomas et al. 2012; Wilson et al. 2013; Zimmermann and Wright 2017). However, no screened study used ipsatized scores for the subscales without reporting the general distress score.

The data set had a nested structure, with occasionally multiple effect sizes for the same mental health domain within one study (e.g., through multiple samples), resulting in a violation of the assumption of independence among effect sizes in meta‐analyses (Cheung 2014; Rosenthal 1986). To address this, a three‐level meta‐analytic model was employed using the guide by Assink and Wibbelink (2016). This allows greater flexibility in analyzing a potential moderator. This approach accounts for variance at three levels: sampling variance (Level 1), within‐study variance (Level 2), and between‐study variance (Level 3).

The three‐level correlational meta‐analysis was conducted using the *metafor* package in *R* (version 4.2‐0; Viechtbauer 2010). The forest plots for each association between interpersonal problems and mental health domain are presented in the Supporting Information. The estimation was based on the restricted maximum likelihood method (Viechtbauer 2005). Before the analysis, correlation coefficients were transformed using Fisher's *z*‐transformation and then back‐transformed for ease of interpretation (Borenstein et al. 2009). The interpretation for the correlation coefficients were as follows: *r* = 0.10 for a small effect size, *r* = 0.30 for a medium, and *r* = 0.50 for a large effect size (Cohen 1988). In addition, Cohen's *d* effect sizes were also provided, with *d* = 0.20 indicating a small effect, *d* = 0.50 a medium effect, and *d* = 0.80 a large effect.

To assess overall heterogeneity, the *Q* and *I* ^*2*^ statistics were used (Higgins et al. 2019; Higgins and Thompson 2002; Huedo‐Medina et al. 2006). The standard *I* ^*2*^ interpretation was applied, with values of 25%, 50%, and 75% indicating low, moderate, and substantial heterogeneity for each level of the model (Assink and Wibbelink 2016).

Publication bias analysis was conducted with an asymmetry test where the variance was entered as a moderator, providing an alternative to Egger's test. In addition, a funnel plot tailored for multilevel meta‐analysis was evaluated, where standard errors (*x*‐axis) are plotted against the effect sizes (y‐axis) using the code provided by Fernández‐Castilla et al. (2020). An asymmetric distribution could indicate potential publication bias. All funnel plots are provided in the Supporting Information S1.

After calculating the pooled effect size and heterogeneity, potential outliers were examined. Model diagnostics, such as Cook's distance and hat values, were performed using the metafor package (Viechtbauer 2010). Additionally, effect sizes below the first or above the third interquartile range were flagged as outliers (Walfish 2006). If an outlier was identified, the analysis was rerun excluding the outlier and compared to the original results.

## 3 Results

### 3.1 Interpersonal Problems and General Psychological Distress

The overall correlation between interpersonal problems and general psychological distress was *r* = 0.565 (*z* = 0.640, *SE* = 0.021, 95% CI [0.597, 0.682]), *t*(28) = 30.755, *p* < 0.001, *s* = 29, *k* = 30, *d* = 1.363. In the Table S1.1 presents the study and effect size descriptives. The *Q* test indicated significant heterogeneity across all effect sizes, *Q*(29) = 148.308, *p* < 0.001. The heterogeneity across the three levels was 19.43% for level 1 (sampling variance), ~ 0% for level 2 (within‐study variance), and 80.57% for level 3 (between‐study variance).

The association remained stable (*r* = 0.559) after removing one potential outlier (Tracey et al. 1996). The alternative to Egger's test showed no indication for a publication bias, as the variance did not significantly account for the variation of the effect size, *F*(1, 28) = 1.733, *p* = 0.199. The forest (S1.2) and funnel plot (S1.3) are provided in the Supporting Information S1.

For the moderator analysis, clinical status (clinical vs. nonclinical) was not a significant moderator of the association between interpersonal problems and general psychological distress, *F*(1, 28) = 2.360, *p* = 0.136.

### 3.2 Interpersonal Problems and Depressive Symptoms

The overall correlation was *r* = 0.473 (*z* = 0.514, *SE* = 0.027, 95% CI [0.459, 0.568]), *t*(35) = 19.242, *p* < 0.001, *s* = 36, *k* = 39, *d* = 1.108. The *Q*‐test was significant *Q*(38) = 428.192, *p* < 0.001. The heterogeneity distributed across the levels as follows: level 1 (sampling variance) = 8.54%, level 2 (within‐study variance) = 42.88%, and level 3 (between‐study variance) = 48.58%. Table S2.1 displays the studies included in the analysis of the association between interpersonal problems and depression.

Three outliers were identified (Akyunus and Gencoz 2016; Fitzpatrick et al. 2020; Ringwald et al. 2024). After their exclusion, the effect size increased slightly to *r* = 0.481 (*z* = 0.525, *SE* = 0.020, 95% CI [0.485, 0.564]). There was no indication of a publication bias based on the analysis, *F*(1, 37) = 2.038, *p* = 0.162. The forest (S2.2) and funnel plot (S2.3) are provided also in the Supporting Information S1.

Clinical status did not significantly account for the variance of the effect sizes, *F*(1, 36) = 0.595, *p* = 0.446. One study was excluded from the moderator analysis, because the sample consisted of clinical and nonclinical individuals (Maxwell et al. 2017).

### 3.3 Interpersonal Problems and Symptoms of Anxiety

The omnibus effect between interpersonal problems and anxiety symptoms was *r* = 0.454 (*z* = 0.490, *SE* = 0.040, 95% CI [0.406, 0.573]), *t*(22) = 12.161, *p* < 0.001, *s* = 23, *k* = 26, *d* = 1.029. The *Q* test indicated significant heterogeneity across all effect sizes, *Q*(25) = 350.148, *p* < 0.001. The heterogeneity was 5.12% for level 1 (sampling variance), 60.41% for level 2 (within‐study variance), and 34.47% for level 3 (between‐study variance). Table S3.1 presents the study and effect size descriptives.

The association remained relatively stable (*r* = 0.432, *z* = 0.463, *SE* = 0.031, 95% CI [0.399, 0.526]) after removing one potential outlier (Kim and Bae 2022). The alternative to Egger's test showed no indication for a publication bias, *F*(1, 24) = 0.412, *p* = 0.527. In the Supporting Information S1, we provide the forest (S3.2) and funnel plot (S3.3).

In regard to the moderator analysis, clinical status was not a significant moderator of the association between interpersonal problems and symptoms of anxiety, *F*(1, 21) = 0.112, *p* = 0.741.

### 3.4 Interpersonal Problems and Positive Emotions

The association between interpersonal problems and positive emotions was *r* = −0.224 (*z* = −0.228, *SE* = 0.066, 95% CI [−0.383, −0.073]), *t*(7) = −3.478, *p* = 0.010, *s* = 8, *k* = 9, *d* = 0.499. Table S4.1 displays the important characteristics of the included studies. The *Q* test for heterogeneity was significant, *Q*(8) = 63.205, *p* < 0.001. The heterogeneity distributed across the three levels as follows: level 1 (sampling variance) = 9.74%, level 2 (within‐study variance) = 6.93%, and level 3 (between‐study variance) = 83.33%.

One outlier was identified (Nicolaou et al. 2023) and removed. The effect size reduced considerably to *r* = −0.175 (*z* = −0.177, *SE* = 0.046, 95% CI [−0.290, −0.064]), *d* = 0.385. Therefore, this study was removed for further analyses. The alternative to Egger's test indicated no significant presence of a publication bias, *F*(1, 6) = 0.006, *p* = 0.942. In the Supporting Information S1, the forest (S4.2) and funnel plot (S4.3) are provided.

Similar to previous results, the association between interpersonal problems and positive emotions was not significantly moderated by the clinical status of the population, *F*(1, 5) = 1.192, *p* = 0.325.

### 3.5 Interpersonal Problems and Negative Emotions

The overall correlation between interpersonal problems and negative emotions was *r* = 0.486 (*z* = 0.531, *SE* = 0.035, 95% CI [0.442, 0.620]), *t*(5) = 15.371, *p* < 0.001, *s* = 6, *k* = 7, *d* = 1.057. Table S5.1 presents the study and effect size descriptives. The *Q* test indicated no significant heterogeneity across all effect sizes, *Q*(6) = 10.637, *p* = 0.100. The heterogeneity across the three levels was 42.96% for level 1 (sampling variance), ~ 0% for level 2 (within‐study variance), and 57.04% for level 3 (between‐study variance).

One outlier was identified (Gurtman 1992b). As this outlier seemed to impact the omnibus effect considerably (*r* = 0.458, *z* = 0.495, *SE* = 0.020, 95% CI [0.440, 0.549], *d* = 1.029), it was removed from further analyses. The alternative to Egger's test showed no indication for a publication bias, *F*(1, 4) = 0.177, *p* = 0.696. In the Supporting Information S1, we provide the forest (S5.2) and funnel plot (S5.3).

Unfortunately, only one remaining study investigated the association between interpersonal problems and negative emotions in a clinical sample. Therefore, no reliable moderator analysis could have been conducted.

### 3.6 Interpersonal Problems and Well‐Being

In the present meta‐analysis, well‐being included the constructs of life satisfaction, quality of life, and mental well‐being. The correlation between interpersonal problems and the domain of well‐being was *r* = −0.372 (*z* = −0.391, *SE* = 0.056, 95% CI [−0.534, −0.247]), *t*(5) = −7.009, *p* < 0.001, *s* = 6, *k* = 7, *d* = 0.805 (see Table S6.1). The *Q*‐test of heterogeneity was significant, *Q*(6) = 51.095, *p* < 0.001. Heterogeneity across the levels was 9.91% for level 1 (sampling variance), 39.43% for level 2 (within‐study variance), and 50.67% for level 3 (between‐study variance).

No outlier was identified, and no publication bias was indicated, *F*(1, 5) = 3.934, *p* = 0.104. The forest (S6.2) and funnel plot (S6.3) are provided in the Supporting Information S1.

Clinical status did not account for significant variance of the effect sizes, *F*(1, 4) = 0.748, *p* = 0.436.

### 3.7 Interpersonal Problems and Stress

For the relationship between interpersonal problems and stress, only two effect sizes were extracted. Therefore, no meta‐analysis could be conducted for this mental health domain (Myung 2023). Descriptively, both correlation coefficients were high (*r* = 0.51; Rakhimov et al. 2023, *r* = 0.55; Ringwald et al. 2024), indicating that higher levels of interpersonal problems were associated with higher perceived stress in both studies, as assessed with the Perceived Stress Scale (PSS; Cohen et al. 1983) in nonclinical samples.

### 3.8 Post‐Hoc Exploratory Moderator Analysis

Given the significant heterogeneity in effect sizes and the lack of a significant moderating effect of clinical status, we conducted a post‐hoc exploratory moderator analysis to further investigate potential sources of variance. Specifically, we examined country, age, and gender distribution (percentage of female participants) as potential moderators. However, none of these factors significantly accounted for the variance in the effect sizes: general psychological distress (country: *p* = 0.638, age: *p* = 0.214, gender: *p* = 0.822), depression (country: *p* = 0.248, age: *p* = 0.827, gender: *p* = 0.416), anxiety (country: *p* = 0.808, age: *p* = 0.847, gender: *p* = 0.348), positive emotions (country: *p* = 0.689, age: *p* = 0.215, gender: *p* = 0.090), negative emotions (country: *p* = 0.808, age: *p* = 0.840, gender: *p* = 0.896), and well‐being (country: *p* = 0.527, age: *p* = 0.906, gender: *p* = 0.073).

## 4 Discussion

The current meta‐analytic review aimed to explore the relationship between interpersonal problems and various aspects of mental health states, including general psychological distress, depressive symptoms, symptoms of anxiety, positive and negative emotions, well‐being, and perceived stress. By systematically consolidating and quantifying findings across 66 studies, this review provides comprehensive, evidence‐based estimates of the strength and consistency of these associations. Drawing from studies with clinical and nonclinical populations, we found robust and strong associations indicating that higher levels of interpersonal problems are linked to poorer mental health across all examined domains. Moreover, the relationships did not differ between clinical and nonclinical samples, and were not significantly moderated by age, gender, or country, indicating that interpersonal problems are consistently associated with mental health across populations and demographic groups.

Throughout this meta‐analytic review, we found that higher levels of interpersonal problems (indicated by the IIP) are associated with increased general psychological distress, more severe symptoms of depression and anxiety, greater negative emotions, fewer positive emotions, lower well‐being, and potentially higher levels of perceived stress. Taken together, these findings suggest that interpersonal problems may reflect a general marker of psychological distress and reduced mental well‐being. This is in line with the idea that different forms of psychological difficulties often share something in common, sometimes described as a general tendency toward mental health problems (Caspi and Moffitt 2018).

Interpersonal problems are therefore likely to co‐occur with a wide range of mental health difficulties. Despite their apparent clinical relevance, they are, with the exception of personality disorders (which is argued to be more of an interpersonal disorder rather than a personality one; Hopwood et al. 2013), not typically considered a core symptom of any specific diagnosis (e.g., American Psychiatric Association 2013). Yet, given their consistent associations with impaired mental health across several domains, interpersonal difficulties may represent an important aspect of the broader psychopathological picture. Often regarded as associated features rather than diagnostic criteria, interpersonal problems may be under‐recognized. This has particular relevance in clinical settings, where interpersonal problems have been linked to poorer therapeutic processes and outcomes (Gómez Penedo et al. 2025; Gómez Penedo and Flückiger 2023; Iovoli et al. 2025).

That interpersonal problems may serve as a general marker of mental health impairment is further supported by the finding that associations did not significantly differ between clinical and nonclinical populations. This suggests that the categorical distinction between these groups may not always be meaningful when examining the role of interpersonal problems in relation to mental health impairment. Instead, the results are more consistent with transdiagnostic and dimensional models of psychopathology (e.g., HiTOP; Kotov et al. 2017), which emphasize the continuity of psychological difficulties.

After considering the general pattern of associations, we now examine how interpersonal problems relate to distinct domains of mental health included in this review. Among all domains, interpersonal problems showed the strongest association with general psychological distress (*r* = 0.565). This may reflect the previously discussed general factor of psychopathology, suggesting that interpersonal problems may be meaningfully connected to it. However, it is also important to consider that some general distress measures, such as the Symptom Checklist‐90‐R, include items or subscales that explicitly reference interpersonal experiences (e.g., interpersonal sensitivity), which may naturally result in stronger correlations with the IIP, especially as some items contain emotionally valenced language. This conceptual overlap could partly inflate the observed effect size in this domain.

Second, interpersonal problems were highly correlated with symptoms of depression (*r* = 0.473) and anxiety (*r* = 0.454). This finding aligns with several theoretical models (e.g., Coyne 1976; Lewinsohn 1974; Segrin 1998), and was to be expected (e.g., Newman et al. 2013). Taken together, these findings suggest that interpersonal problems are strongly associated with internalizing symptoms of psychopathology. This points to a close connection between interpersonal and intrapersonal domains of distress. While the effect sizes are based on cross‐sectional data and cannot clarify directionality and causality, it is notable that difficulties in relating to others are closely tied to internal psychological suffering. This interplay may reflect how maladaptive interpersonal behavior can shape emotional experiences, or how internal states influence how individuals engage in relationships. In other words, interpersonal experiences may inform intrapersonal distress, and vice versa.

Lastly, interpersonal problems were also significantly related to emotional experiences, showing associations with higher negative emotions (*r* = 0.486), lower positive emotions (*r* = −0.224), and general well‐being (*r* = −0.372). This pattern may reflect a more general emotional tone of distress associated with interpersonal difficulties. Moreover, previous studies suggest that individuals with interpersonal problems are more likely to struggle with effective emotion regulation (Christ et al. 2019; Dimaggio et al. 2017; Garofalo et al. 2017), which may contribute to this emotional profile. Moreover, since interpersonal relationships often serve as important sources of emotional support and regulation (e.g., Barthel et al. 2018), the perception of relationships as distressing may deprive individuals of key emotion regulation resources.

### 4.1 Limitations

Several limitations should be considered when interpreting the results of this meta‐analytic review. First and foremost, we mentioned above that the presented analysis is based on correlational data from observational or interventional studies. This inherently limits the ability to draw causal inferences about the reciprocal relationship between interpersonal problems and specific mental health domains. To investigate causal pathways and directionality of the effects, longitudinal studies and experimental designs are needed. Second, studies from Eastern cultures are highly underrepresented in the meta‐analysis. This cultural bias necessitates caution when generalizing the findings into non‐Western contexts, as interpersonal relationships and their relation to mental health can vary across cultures (Burleson 2003). Third, we exclusively relied on the IIP as an indicator for interpersonal problems, and self‐report measures to operationalize mental health domains. Therefore, common biases like social desirability or recall might affect the accuracy of the reported constructs. Fourth, we focused primarily on the general distress associated with interpersonal problems. The IIP would offer the differentiation of subtypes of interpersonal problems that may have distinct impacts on various mental health outcomes. Future reviews could include subtypes to provide a more detailed understanding of the relationship between interpersonal problems and mental health. Fifth, we used an inclusion criterion that required studies to have at least 50 participants to ensure the inclusion of studies. However, there is evidence suggesting that correlation coefficients stabilize at a sample size of approximately 250 (Schönbrodt and Perugini 2013), which is relatively high for studies in clinical populations. Therefore, some of the included studies may not have had sufficiently large sample sizes to provide stable correlation estimates. Lastly, the mental health domain of well‐being in our analysis was composed of three interrelated, yet slightly different constructs: life satisfaction, quality of life, and mental well‐being. It is important to note that some of these indicators also encompass physiological or other nonmental aspects (e.g., job satisfaction, educational status, wealth, etc.). Consequently, this approach might have confounded the interpretation of the relationship between interpersonal problems and well‐being.

### 4.2 Future Directions

Our findings highlight the value of adopting a more comprehensive approach to mental health by incorporating interpersonal problems into transdiagnostic models of psychopathology. One promising avenue for this is the integration of interpersonal problems into the network approach to psychopathology (Borsboom 2017; McNally 2016). Networks conceptualize mental disorders as complex systems of interacting elements rather than discrete entities (Bringmann et al. 2022). Incorporating interpersonal problems into this network model offers a valuable avenue for future research. By treating them as nodes within the broader network of mental health symptoms, researchers can investigate how these interpersonal issues (or other non‐symptom nodes) interact with symptoms (e.g., Iovoli et al. 2024). Longitudinal studies, in particular, could provide insights into the temporal dynamics and causal relationships between interpersonal problems and various mental health indicators. Additionally, given the mentioned underrepresentation of studies in non‐Western cultures, future research should place more emphasis on cross‐cultural investigations of the relationship between interpersonal problems and aspects of mental health. Future research could also examine variability in interpersonal behavior. While our review focused on trait‐like interpersonal problems, recent studies suggest that fluctuations in interpersonal behavior may represent another meaningful marker of psychological dysfunction (e.g., Rappaport et al. 2014; Ringwald et al. 2022, 2023; Wright et al. 2015).

## 5 Conclusion

In conclusion, the findings of this meta‐analytic review underscore the close interrelations between interpersonal problems and various aspects of mental health. Our results highlight that higher levels of interpersonal problems are strongly associated with increased general psychological distress, more severe symptoms of depression and anxiety, greater negative emotions, fewer positive emotions, lower well‐being, and potentially higher perceived stress. Notably, these associations were consistent across both clinical and nonclinical populations, indicating the universal relevance of interpersonal problems and their relation to mental health. Future research should further explore causal pathways and cultural differences to refine our understanding of this relationship.

## Ethics Statement

Ethical approval was not necessary for this study, as it is a meta‐analysis based solely on peer‐reviewed, published work. However, the study was preregistered on PROSPERO (CRD42024563448).

## Conflicts of Interest

The authors declare no conflicts of interest.

## Supporting information


**Table S1.1:** Study and Effect Size Characteristics for Studies Assessing General Psychological Distress. **Table S2.1:** Study and Effect Size Characteristics for Studies Assessing Depressive Symptoms. **Table S3.1:** Study and Effect Size Characteristics for Studies Assessing Symptoms of Anxiety. **Table S4.1:** Study and Effect Size Characteristics for Studies Assessing Positive Emotions. **Table S5.1:** Study and Effect Size Characteristics for Studies Assessing Negative Emotions. **Table S6.1:** Study and Effect Size Characteristics for Studies Assessing Well‐Being. **S1.2:** Forest Plot. **S1.3:** Funnel Plot. **S2.2:** Forest Plot. **S2.3:** Funnel Plot. **S3.2:** Forest Plot. **S3.3:** Funnel Plot. **S4.2:** Forest Plot. **S4.3:** Funnel Plot. **S5.2:** Forest Plot. **S5.3:** Funnel Plot. **S6.2:** Forest Plot. **S6.3:** Funnel Plot.

## Data Availability

The data (as well as the literature search results and included studies) for this meta‐analysis will be made publicly available on Open Science Framework (https://osf.io/tge69/).
