# Supplementary material for: Interpersonal Problems and Their Mental Health Correlates: A Meta‐Analytic Review
Source: J Clin Psychol. 2025 Aug 4;81(11):1046–56. doi: 10.1002/jclp.70022 (PMC12501831; doi:10.1002/jclp.70022)
Supplement: Supplementary file 1 — Table S1.1: Study and Effect Size Characteristics for Studies Assessing General Psychological Distress. Table S2.1: Study and Effect Size Characteristics for Studies Assessing Depressive Symptoms. Table S3.1: Study and Effect Size Characteristics for Studies Assessing Symptoms of Anxiety. Table S4.1: Study and Effect Size Characteristics for Studies Assessing Positive Emotions. Table S5.1: Study and Effect Size Characteristics for Studies Assessing Negative Emotions. Table S6.1: Study and Effect Size Characteristics for Studies Assessing Well‐Being. S1.2: Forest Plot. S1.3: Funnel Plot. S2.2: Forest Plot. S2.3: Funnel Plot. S3.2: Forest Plot. S3.3: Funnel Plot. S4.2: Forest Plot. S4.3: Funnel Plot. S5.2: Forest Plot. S5.3: Funnel Plot. S6.2: Forest Plot. S6.3: Funnel Plot. [file JCLP-81-1046-s001.docx]

**Supplementary Material to**

**“Interpersonal Problems and Their Mental Health Correlates: A Meta-Analytic Review”**

Included studies (s = 66):

Akyunus, M., & Gencoz, T. (2016). Psychometric Properties of the Inventory of Interpersonal Problems-Circumplex Scales Short Form: A Reliability and Validity Study. *Dusunen Adam: The Journal of Psychiatry and Neurological Sciences*, 36–48. https://doi.org/10.5350/DAJPN2016290104

Ansell, E. B., Grilo, C. M., & White, M. A. (2012). Examining the interpersonal model of binge eating and loss of control over eating in women. *International Journal of Eating Disorders*, *45*(1), 43–50. https://doi.org/10.1002/eat.20897

Atlas, L., & Zweig, R. (2022). The Doctor-Patient Relationship, Personality, Mood and Functioning in Older Adults. *Professional Psychology-Research and Practice* *53*(5), 436–445. https://doi.org/10.1037/pro0000419

Barkham, M., Hardy, G. E., & Startup, M. (1994). The structure, validity and clinical relevance of the Inventory of Interpersonal Problems. *British Journal of Medical Psychology*, *67*(2), 171–185. https://doi.org/10.1111/j.2044-8341.1994.tb01784.x

Benecke, C., Vogt, T., Bock, A., Koschier, A., & Peham, D. (2008). Emotionserleben und Emotionsregulation und ihr Zusammenhang mit psychischer Symptomatik [Emotional Experience and Emotion Regulation and Their Relationship with Psychological Symptoms]. *PPmP - Psychotherapie · Psychosomatik · Medizinische Psychologie*, *58*(09/10), 366–370. https://doi.org/10.1055/s-2007-986319

Brugnera, A., Carlucci, S., Compare, A., & Tasca, G. A. (2019). Persistence of friendly and submissive interpersonal styles among those with binge‐eating disorder: Comparisons with matched controls and outcomes after group therapy. *Clinical Psychology & Psychotherapy*, *26*(5), 603–615. https://doi.org/10.1002/cpp.2385

Cheavens, J. S., Strunk, D. R., & Chriki, L. (2012). A Comparison of Three Theoretically Important Constructs: What Accounts For Symptoms of Borderline Personality Disorder? *Journal of Clinical Psychology*, *68*(4), 477–486. https://doi.org/10.1002/jclp.20870

Chen, L., Yan, Z., Tang, W., Yang, F., Xie, X., & He, J. (2016). Mobile phone addiction levels and negative emotions among Chinese young adults: The mediating role of interpersonal problems. *Computers in Human Behavior*, *55*, 856–866. https://doi.org/10.1016/j.chb.2015.10.030

Chiesa, M., Larsen-Paya, M., Martino, M., & Trinchieri, M. (2016). The relationship between childhood adversity, psychiatric disorder and clinical severity: Results from a multi-centre study. *Psychoanalytic Psychotherapy*, *30*(1), 79–95. https://doi.org/10.1080/02668734.2016.1145131

Connolly Gibbons, M. B., Crits-Christoph, P., De La Cruz, C., Barber, J. P., Siqueland, L., & Gladis, M. (2003). Pretreatment expectations, interpersonal functioning, and symptoms in the prediction of the therapeutic alliance across supportive-expressive psychotherapy and cognitive therapy. *Psychotherapy Research*, *13*(1), 59–76. https://doi.org/10.1093/ptr/kpg007

Cox, D. W., Kealy, D., Kahn, J. H., McCloskey, K. D., Joyce, A. S., & Ogrodniczuk, J. S. (2020). Depression symptoms’ impact on personality disorder treatment: Depression symptoms amplifying the interpersonal benefits of negative-affect expression. *Journal of Affective Disorders*, *272*, 318–325. https://doi.org/10.1016/j.jad.2020.03.133

Dally, A., Falck, O., Ferrari, T., Leichsenring, F., Rabung, S., & Streeck, U. (2005). Soziale Ängste in einer klinischen Population [Social Anxiety in a Clinical Population]. *PPmP - Psychotherapie · Psychosomatik · Medizinische Psychologie*, *55*(3/4), 169–176. https://doi.org/10.1055/s-2004-834631

De Panfilis, C., Meehan, K. B., Cain, N. M., & Clarkin, J. F. (2013). The relationship between effortful control, current psychopathology and interpersonal difficulties in adulthood. *Comprehensive Psychiatry*, *54*(5), 454–461. https://doi.org/10.1016/j.comppsych.2012.12.015

Desmet, M., Van Hoorde, H., Verhaeghe, P., Meganck, R., Vanheule, S., & Van Den Abeele, T. (2008). Interpersonal profiles and neurotic symptoms: Are they associated with each other? *Psychoanalytic Psychology*, *25*(2), 342–355. https://doi.org/10.1037/0736-9735.25.2.342

Dimaggio, G., MacBeth, A., Popolo, R., Salvatore, G., Perrini, F., Raouna, A., Osam, C. S., Buonocore, L., Bandiera, A., & Montano, A. (2018). The problem of overcontrol: Perfectionism, emotional inhibition, and personality disorders. *Comprehensive Psychiatry*, *83*, 71–78. https://doi.org/10.1016/j.comppsych.2018.03.005

Dinger, U., Zimmermann, J., Masuhr, O., & Spitzer, C. (2017). Therapist effects on outcome and alliance in inpatient psychotherapy: The contribution of patients’ symptom severity. *Psychotherapy*, *54*(2), 167–174. https://doi.org/10.1037/pst0000059

Dobson, K. S., McLarnon, M. J. W., Pandya, K., & Pusch, D. (2021). A latent profile analysis of adverse childhood experiences and adult health in a community sample. *Child Abuse & Neglect*, *114*, 104927. https://doi.org/10.1016/j.chiabu.2020.104927

Edwards, K. M., Probst, D. R., Rodenhizer-Stämpfli, K. A., Gidycz, C. A., & Tansill, E. C. (2014). Multiplicity of Child Maltreatment and Biopsychosocial Outcomes in Young Adulthood: The Moderating Role of Resiliency Characteristics Among Female Survivors. *Child Maltreatment*, *19*(3–4), 188–198. https://doi.org/10.1177/1077559514543354

Erickson, T. M., Lewis, J. A., Crouch, T. A., Singh, N. B., & Cummings, M. H. (2023). Interpersonal contrast avoidance as a mechanism for the maintenance of worry. *Journal of Anxiety Disorders*, *94*, 102678. https://doi.org/10.1016/j.janxdis.2023.102678

Fitzpatrick, O. M., Whelen, M. L., Falkenström, F., & Strunk, D. R. (2020). Who benefits the most from cognitive change in cognitive therapy of depression? A study of interpersonal factors. *Journal of Consulting and Clinical Psychology*, *88*(2), 128–136. https://doi.org/10.1037/ccp0000463

Flemming, E., Lübke, L., Müller, S., Lorenzen, V., & Spitzer, C. (2022). Bindungsdimensionen im Erwachsenenalter – beziehungsspezifische Unterschiede und ihre Zusammenhänge mit psychischer Gesundheit [Attachment Dimensions in Adulthood – Relationship-Specific Differences and Their Connections to Mental Health]. *Die Psychotherapie*, *67*(6), 493–500. https://doi.org/10.1007/s00278-022-00610-0

Gómez Penedo, J. M., Schwartz, B., Deisenhofer, A.-K., Rubel, J., Babl, A. M., & Lutz, W. (2021). Interpersonal clarification effects in Cognitive-Behavioral Therapy for depression and how they are moderated by the therapeutic alliance. *Journal of Affective Disorders*, *279*, 662–670. https://doi.org/10.1016/j.jad.2020.10.043

González-Cifuentes, C. E., & Ruiz, F. J. (2022). Psychometric properties of the Inventory of Interpersonal Problems-64 in Colombia. *International Journal of Psychology & Psychological Therapy*, *22*(2), 223–234.

Gude, T., Vaglum, P., Tyssen, R., Ekeberg, O., Hem, E., Rovik, J. O., Finset, K., & Gronvold, N. T. (2005). Identification with the role of doctor at the end of medical school: A nationwide longitudinal study. *Medical Education*, *39*(1), 66–74. https://doi.org/10.1111/j.1365-2929.2004.02034.x

Gurtman, M. B. (1992a). Construct validity of interpersonal personality measures: The interpersonal circumplex as a nomological net. *Journal of Personality and Social Psychology*, *63*(1), 105–118.

Hayden, M. C., Müllauer, P. K., Gaugeler, R., Senft, B., & Andreas, S. (2019). Mentalization as Mediator between Adult Attachment and Interpersonal Distress. *Psychopathology*, *52*(1), 10–17. https://doi.org/10.1159/000496499

Hess, T. R., Rohlfing, J. E., Hardy, A. O., Glidden-Tracey, C., & Tracey, T. J. G. (2010). An Examination of the “Interpersonalness” of the Outcome Questionnaire. *Assessment*, *17*(3), 396–399. https://doi.org/10.1177/1073191110369761

Horowitz, L. M., Rosenberg, S. E., Baer, B. A., Ureño, G., & Villaseñor, V. S. (1988). Inventory of interpersonal problems: Psychometric properties and clinical applications. *Journal of Consulting and Clinical Psychology*, *56*(6), 885–892. https://doi.org/10.1037/0022-006X.56.6.885

Huprich, S. K., Lengu, K., & Evich, C. (2016). Interpersonal Problems and Their Relationship to Depression, Self-Esteem, and Malignant Self-Regard. *Journal of Personality Disorders*, *30*(6), 742–761. https://doi.org/10.1521/pedi_2015_29_227

Juen, F., Vierl, L., & Hörz-Sagstetter, S. (2024). Patientencharakteristika an einer psychodynamischen Ausund Weiterbildungsambulanz [Patient characteristics at a psychodynamic training and further education clinic]. *Zeitschrift Für Psychosomatische Medizin Und Psychotherapie*, OA1. https://doi.org/10.13109/zptm.2024.70.oa3

Kim, S.-S., & Bae, S.-M. (2022). Social Anxiety and Social Networking Service Addiction Proneness in University Students: The Mediating Effects of Experiential Avoidance and Interpersonal Problems. *Psychiatry Investigation*, *19*(6), 462–469. https://doi.org/10.30773/pi.2021.0298

Koppelberg, P., Kersting, A., & Suslow, T. (2023). Alexithymia and interpersonal problems in healthy young individuals. *BMC Psychiatry*, *23*(1), 688. https://doi.org/10.1186/s12888-023-05191-z

Kvarstein, E. H., & Karterud, S. (2012). Large Variations of Global Functioning over Five Years in Treated Patients with Personality Traits and Disorders. *Journal of Personality Disorders*, *26*(2), 141–161. https://doi.org/10.1521/pedi.2012.26.2.141

Lambert, M. J., Hannöver, W., Nisslmüller, K., Richard, M., & Kordy, H. (2002). Fragebogen zum Ergebnis von Psychotherapie: *Zeitschrift für Klinische Psychologie und Psychotherapie*, *31*(1), 40–46. https://doi.org/10.1026/0084-5345.31.1.40

Lejuez, C. W., Daughters, S. B., Nowak, J. A., Lynch, T., Rosenthal, M. Z., & Kosson, D. (2003). Examining the inventory of interpersonal problems as a tool for conducting analogue studies of mechanisms underlying Borderline Personality Disorder. *Journal of Behavior Therapy and Experimental Psychiatry*, *34*(3–4), 313–324. https://doi.org/10.1016/j.jbtep.2003.11.002

Lilliengren, P., Falkenström, F., Sandell, R., Mothander, P. R., & Werbart, A. (2015). Secure attachment to therapist, alliance, and outcome in psychoanalytic psychotherapy with young adults. *Journal of Counseling Psychology*, *62*(1), 1–13. psyh. https://doi.org/10.1037/cou0000044

Liu, Y., Hopwood, C. J., Pincus, A. L., Zhou, B., Yang, J., Bai, S., & Yang, B. (2024). Interpersonal Problem Profiles of Personality and Psychopathology Constructs in Chinese Undergraduates and Offenders. *Assessment*, 10731911241241495. https://doi.org/10.1177/10731911241241495

Lo Coco, G., Mannino, G., Salerno, L., Oieni, V., Di Fratello, C., Profita, G., & Gullo, S. (2018). The Italian Version of the Inventory of Interpersonal Problems (IIP-32): Psychometric Properties and Factor Structure in Clinical and Non-clinical Groups. *Frontiers in Psychology*, *9*, 341. https://doi.org/10.3389/fpsyg.2018.00341

Lorentzen, S., Ruud, T., Fjeldstad, A., & Høglend, P. A. (2015). Personality disorder moderates outcome in short‐ and long‐term group analytic psychotherapy: A randomized clinical trial. *British Journal of Clinical Psychology*, *54*(2), 129–146. https://doi.org/10.1111/bjc.12065

Malat, J., & Turner, N. E. (2013). Characteristics of Outpatients in an Addictions Clinic for Co‐occurring Disorders. *The American Journal on Addictions*, *22*(3), 297–301. https://doi.org/10.1111/j.1521-0391.2012.12001.x

Malivoire, B. L., & Koerner, N. (2022). Interpersonal dysfunction in individuals high in chronic worry: Relations with interpersonal problem-solving. *Behavioural and Cognitive Psychotherapy*, *50*(2), 142–157. https://doi.org/10.1017/S1352465821000436

Maxwell, H., Tasca, G. A., Grenon, R., Faye, M., Ritchie, K., Bissada, H., & Balfour, L. (2017). The role of coherence of mind and reflective functioning in understanding binge-eating disorder and co-morbid overweight. *Attachment & Human Development*, *19*(4), 407–424. https://doi.org/10.1080/14616734.2017.1318934

McEvoy, P. M., Burgess, M. M., Page, A. C., Nathan, P., & Fursland, A. (2013). Interpersonal problems across anxiety, depression, and eating disorders: A transdiagnostic examination. *British Journal of Clinical Psychology*, *52*(2), 129–147. https://doi.org/10.1111/bjc.12005

Müller, S., Wendt, L. P., Spitzer, C., Masuhr, O., Back, S. N., & Zimmermann, J. (2022). A Critical Evaluation of the Reflective Functioning Questionnaire (RFQ). *Journal of Personality Assessment*, *104*(5), 613–627. https://doi.org/10.1080/00223891.2021.1981346

Natoli, A. P., Nelson, S. M., Lengu, K. J., & Huprich, S. K. (2016). Sensitivity to criticism differentially mediates the relationship between interpersonal problems and state and trait depression: Sensitivity to criticism in depression. *Personality and Mental Health*, *10*(4), 293–304. https://doi.org/10.1002/pmh.1338

Nicolaou, S., Goldberg, S. F., Michael, K. M., & Berenson, K. R. (2023). Responses to validating versus reframing support strategies as a function of borderline personality features and interpersonal problems. *Cogent Psychology*, *10*(1), 2146280. https://doi.org/10.1080/23311908.2022.2146280

Nordahl, H., Hjemdal, O., & Wells, A. (2021). Metacognitive Beliefs Uniquely Contribute to Interpersonal Problems: A Test Controlling for Adult Attachment, Big-5 Personality Traits, Anxiety, and Depression. *FRONTIERS IN PSYCHOLOGY*, *12*. https://doi.org/10.3389/fpsyg.2021.694565

Nordahl, H., Strand, E. R., Hjemdal, O., & Nordahl, H. M. (2024). Is meta-worry relevant to interpersonal problems? Testing the metacognitive model of generalized anxiety disorder in an analogue- and a clinical sample of GAD. *Cognitive Behaviour Therapy*, *53*(5), 455–466. https://doi.org/10.1080/16506073.2024.2331191

Prout, T. A., Magaldi, D., Kim, E. E., & Cha, J. (2021). Christian therapists and their clients. *Spirituality in Clinical Practice*, *8*(1), 1–15. https://doi.org/10.1037/scp0000238

Rakhimov, A., Ong, J., Realo, A., & Tang, N. K. Y. (2023). Being kind to self is being kind to sleep? A structural equation modelling approach evaluating the direct and indirect associations of self-compassion with sleep quality, emotional distress and mental well-being. *Current Psychology*, *42*(16), 14092–14105. https://doi.org/10.1007/s12144-021-02661-z

Ringwald, W. R., Woods, W. C., & Wright, A. G. C. (2024). Comparing the Diagnostic and Statistical Manual of Mental Disorders, fifth edition, personality disorder models scored from the same interview. *Personality Disorders: Theory, Research, and Treatment*, *15*(5), 371–378. https://doi.org/10.1037/per0000663

Ryum, T., Vogel, P. A., Walderhaug, E. P., & Stiles, T. C. (2015). The role of self‐image as a predictor of psychotherapy outcome. *Scandinavian Journal of Psychology*, *56*(1), 62–68. https://doi.org/10.1111/sjop.12167

Salerno, L., Lo Coco, G., Gullo, S., Iacoponelli, R., Caltabiano, M. L., & Ricciardelli, L. A. (2015). Self‐esteem mediates the associations among negative affect, body disturbances, and interpersonal problems in treatment‐seeking obese individuals. *Clinical Psychologist*, *19*(2), 85–95. https://doi.org/10.1111/cp.12036

Schmitz, N., Hartkamp, N., Kiuse, J., Franke, G. H., Reister, G., & Tress, W. (2000). The symptom check-list-90-R (SCL-90-R): A German validation study. *Quality of Life Research*, *9*(2), 185–193. https://doi.org/10.1023/A:1008931926181

Schürmann‐Vengels, J., Teismann, T., Margraf, J., & Willutzki, U. (2022). Patients’ self‐perceived strengths increase during treatment and predict outcome in outpatient cognitive behavioral therapy. *Journal of Clinical Psychology*, *78*(12), 2427–2445. https://doi.org/10.1002/jclp.23352

Shin, K. E., & Newman, M. G. (2019). Self- and other-perceptions of interpersonal problems: Effects of generalized anxiety, social anxiety, and depression. *Journal of Anxiety Disorders*, *65*, 1–10. https://doi.org/10.1016/j.janxdis.2019.04.005

Spitzer, C., Siebel-Jürges, U., Barnow, S., Grabe, H. J., & Freyberger, H. J. (2005). Alexithymia and Interpersonal Problems. *Psychotherapy and Psychosomatics*, *74*(4), 240–246. https://doi.org/10.1159/000085148

Sun, Q.-W., Wang, C. D. C., & Jiang, G.-R. (2017). Culture-Based Emotional Working Models of Attachment, Western-Based Attachment, and Psychosocial Functioning of Chinese Young Adults. *International Perspectives in Psychology*, *6*(4), 195–208. https://doi.org/10.1037/ipp0000075

Tracey, T. J. G., Rounds, J., & Gurtman, M. (1996). Examination of the General Factor with the Interpersonal Circumplex Structure: Application to the Inventory of Interpersonal Problems. *Multivariate Behavioral Research*, *31*(4), 441–466. https://doi.org/10.1207/s15327906mbr3104_3

Vierl, L., Juen, F., Benecke, C., & Hörz‐Sagstetter, S. (2023). Exploring the associations between psychodynamic constructs and psychopathology: A network approach. *Personality and Mental Health*, *17*(1), 40–54. https://doi.org/10.1002/pmh.1559

Webb, J. R., Hirsch, J. K., Visser, P. L., & Brewer, K. G. (2013). Forgiveness and Health: Assessing the Mediating Effect of Health Behavior, Social Support, and Interpersonal Functioning. *The Journal of Psychology*, *147*(5), 391–414. https://doi.org/10.1080/00223980.2012.700964

Wei, M., Heppner, P. P., & Mallinckrodt, B. (2003). Perceived coping as a mediator between attachment and psychological distress: A structural equation modeling approach. *Journal of Counseling Psychology*, *50*(4), 438–447. https://doi.org/10.1037/0022-0167.50.4.438

Wei, M., Mallinckrodt, B., Arterberry, B. J., Liu, S., & Wang, K. T. (2021). Latent profile analysis of interpersonal problems: Attachment, basic psychological need frustration, and psychological outcomes. *Journal of Counseling Psychology*, *68*(4), 467–488. https://doi.org/10.1037/cou0000551

Wei, M., Vogel, D. L., Ku, T.-Y., & Zakalik, R. A. (2005). Adult Attachment, Affect Regulation, Negative Mood, and Interpersonal Problems: The Mediating Roles of Emotional Reactivity and Emotional Cutoff. *Journal of Counseling Psychology*, *52*(1), 14–24. https://doi.org/10.1037/0022-0167.52.1.14

Wiseman, H., Raz, A., & Sharabany, R. (2007). *Depressive personality styles and interpersonal problems in young adults with difficulties in establishing long-term romantic relationships*. Israel Journal of Psychiatry and Related Sciences, *44*(4), 280–291.

Zimmermann, J., Wolf, M., Bock, A., Peham, D., & Benecke, C. (2013). The way we refer to ourselves reflects how we relate to others: Associations between first-person pronoun use and interpersonal problems. *Journal of Research in Personality*, *47*(3), 218–225. https://doi.org/10.1016/j.jrp.2013.01.008

**S1 Interpersonal Problems and General Distress**

**S1.1** **Table** Study and Effect Size Characteristics for Studies Assessing General Psychological Distress

|  | Study characteristic | | | | | |  | Effect size  characteristics | |
| --- | --- | --- | --- | --- | --- | --- | --- | --- | --- |
| Publication | Country | Population | Clinical  status | Mean age | Female  % | Mental health measure |  | Correlation coefficient (*r*) | Sample size |
| Akyunus & Gencoz (2016)a | Turkey | community volunteers | non-clinical | 26,85 | 68,34 | BSI |  | 0,52 | 988 |
| Barkham et al. (1994)a | UK | seeking psychotherapy | clinical | 38,96 | 49,1 | SCL-90R |  | 0,58 | 143 |
| Chiesa et al. (2016) | UK | mixed patient sample | clinical | 37,5 | 64,4 | BSI |  | 0,6 | 1136 |
| De Panfilis et al. (2013) | USA | undergraduates | non-clinical | 21,5 | 82,5 | BSI |  | 0,47 | 240 |
| Desmet et al. (2008) | Belgium | undergraduates | non-clinical | 23,35 | 88,08 | SCL-90R |  | 0,57 | 151 |
| Dimaggio et al. (2018)b | Italy | treatment-seeking outpatients | clinical | 35,8 | 54,3 | SCL-90R |  | 0,543 | 578 |
| Dinger et al. (2017) | Germany | inpatients | clinical | 35,6 | 63,6 | SCL-90R |  | 0,63 | 3051 |
| Edwards et al. (2014) | USA | undergraduates | non-clinical | 18,74 | 100 | BSI |  | 0,54 | 765 |
| Flemming et al. (2022) | Germany | community volunteers | non-clinical | 33 | 86 | SCL-K9 |  | 0,57 | 622 |
| Gómez Penedo et al. (2021) | Germany | patients with major depressive disorder | clinical | 37,9 | 60,7 | HSCL-11 |  | 0,51 | 621 |
| González-Cifuentes & Ruiz (2022)a | Colombia | community volunteers | non-clinical | 34 | 55,2 | GHQ-12 |  | 0,39 | 701 |
| Gude et al. (2005) | Norway | undergraduates | non-clinical | 21,7 | 59 | GHQ-28 |  | 0,4 | 236 |
| Hayden et al. (2019) | Austria | patients | clinical | 43,95 | 52,81 | BSI-18 |  | 0,636 | 89 |
| Hess et al. (2010) | USA | community clients | clinical | - | 62,81 | OQ45-SD |  | 0,53 | 121 |
| Horowitz et al. (1988) | USA | treatment-seeking outpatients | clinical | 32,7 | 86,41 | SCL-90R |  | 0,64 | 103 |
| Kvarstein & Karterud (2012) | Norway | patients with personality disorder | clinical | 34 | 75 | SCL-90R |  | 0,62 | 352 |
| Lambert et al. (2002) | Germany | community volunteers | non-clinical | 35,6 | 56 | OQ45-SD |  | 0,64 | 232 |
| Lilliengren et al. (2015) | Sweden | young patients | clinical | 22 | 80 | SCL-90R |  | 0,64 | 70 |
| Lo Coco et al. (2018)a | Italy | community volunteers | non-clinical | 30,32 | 68 | OQ-45 |  | 0,582 | 296 |
| Lo Coco et al. (2018)b | Italy | patients | clinical | 39,81 | 83 | OQ-45 |  | 0,538 | 601 |
| Lorentzen et al. (2015) | Norway | outpatients | clinical | 38,4 | 63 | SCL-90R |  | 0,55 | 167 |
| Malat & Turner (2013) | Canada | treatment-seeking outpatients | clinical | 43,8 | 53 | BSI |  | 0,58 | 131 |
| Müller et al. (2022)a | Germany | inpatients | clinical | 34 | 64 | BSI |  | 0,63 | 861 |
| Prout et al. (2021) | USA | treatment-seeking outpatients | clinical | 29,88 | 75,4 | SCL-10R |  | 0,53 | 142 |
| Ryum et al. (2015) | Norway | patients | clinical | 34,7 | 71,8 | SCL-90R |  | 0,59 | 170 |
| Schmitz et al. (2000)a | Germany | outpatients | clinical | 37 | 60,4 | SCL-90R |  | 0,63 | 1886 |
| Schürmann‐Vengels et al. (2022) | Germany | outpatients | clinical | 42,49 | 56,44 | BSI |  | 0,41 | 185 |
| Spitzer et al. (2005) | Germany | inpatients | clinical | 39,5 | 72,5 | SCL-90R |  | 0,58 | 149 |
| Tracey et al. (1996)a | USA | undergraduates | non-clinical | - | 58,1 | BSI |  | 0,75 | 105 |
| Webb et al. (2013) | USA | undergraduates | non-clinical | 22,11 | 74 | DASS |  | 0,53 | 363 |

*Note*. *k* = 30; BSI = Brief Symptom Inventory, SCL = Symptom Checklist, OQ-45(-SD) = Outcome Questionnaire (SD represents the symptom distress subscale), HSCL = Hopkins Symptom Checklist, GHQ = General Health Questionnaire, DASS = Depression Anxiety Stress Scale.

**S1.2 Forest Plot**

**S1.3 Funnel Plot**

**S2 Interpersonal Problems and Symptoms of Depression**

**S2.1 Table** Study and Effect Size Characteristics for Studies Assessing Depressive Symptoms

|  | Study characteristic | | | | | |  | Effect size  characteristics | |
| --- | --- | --- | --- | --- | --- | --- | --- | --- | --- |
| Publication | Country | Population | Clinical  status | Mean age | Female  % | Mental health measure |  | Correlation coefficient (*r*) | Sample size |
| Akyunus & Gencoz (2016)b | Turkey | community volunteers | non-clinical | 26,85 | 68,34 | BSI-Depression |  | 0,73 | 988 |
| Ansell et al. (2012) | USA | community volunteers | non-clinical | 35 | 100 | BDI |  | 0,59 | 350 |
| Atlas & Zweig (2022) | USA | older adults | non-clinical | 70,73 | 70 | GDS |  | 0,383 | 170 |
| Barkham et al. (1994)b | UK | seeking psychotherapy | clinical | 38,96 | 49,1 | SCL-90R-Depression |  | 0,28 | 143 |
| Brugnera et al. (2019) | Canada | women with BED | clinical | 44,42 | 100 | BDI-II |  | 0,3 | 101 |
| Cheavens et al. (2012) | USA | community volunteers and undergraduates | non-clinical | 24,93 | 63,2 | CES-D |  | 0,6 | 538 |
| Chen et al. (2016)a | China | undergraduates (possible mobile phone addicts) | non-clinical | 20,12 | 57,41 | CES-D |  | 0,57 | 634 |
| Connolly Gibbons et al. (2003)a | USA | patients | clinical | 37,27 | 49 | BDI |  | 0,44 | 141 |
| Cox et al. (2020) | Canada | patients with a personality disorder | clinical | 38,91 | 66,9 | BSI-Depression |  | 0,5 | 239 |
| Dimaggio et al. (2018)a | Italy | treatment-seeking outpatients | clinical | 35,8 | 54,3 | BDI-II |  | 0,446 | 578 |
| Dobson et al. (2021)b | Canada | community volunteers | non-clinical | 44,13 | - | PHQ-9 |  | 0,44 | 3932 |
| Erickson et al. (2023)b | USA | undergraduates | non-clinical | 19,56 | 76 | CES-D |  | 0,55 | 92 |
| Fitzpatrick et al. (2020) | USA | patients with major depressive disorder | clinical | 31,7 | 60 | BDI-II |  | 0 | 126 |
| Gómez Penedo et al. (2021)a | Germany | patients with major depressive disorder | clinical | 37,9 | 60,7 | PHQ-9 |  | 0,46 | 621 |
| Huprich et al. (2016) | USA | undergraduates | non-clinical | - | 72,4 | BDI-II |  | 0,41 | 840 |
| Koppelberg et al. (2023)b | Germany | community volunteers | non-clinical | 23,97 | 50 | BDI-II |  | 0,51 | 200 |
| Lejuez et al. (2003)a | USA | university students | non-clinical | 23,5 | 59 | CES-D |  | 0,53 | 78 |
| Lejuez et al. (2003)b | USA | inpatients | clinical | 40,6 | 42 | CES-D |  | 0,5 | 78 |
| Liu et al. (2024)a | China | undergraduates | non-clinical | 20,26 | 59,5 | PHQ-9 |  | 0,42 | 511 |
| Liu et al. (2024)e | China | male offenders | non-clinical | 40,87 | 0 | PHQ-9 |  | 0,51 | 979 |
| Maxwell et al. (2017) | Canada | women with and without BED | - | 45,1 | 100 | BDI-II |  | 0,62 | 194 |
| McEvoy et al. (2013)a | Australia | anxiety and depression patients | clinical | 36,42 | 65 | BDI-II |  | 0,47 | 457 |
| McEvoy et al. (2013)d | Australia | eating disorder patients | clinical | 25,65 | 98,2 | DASS-D |  | 0,6 | 334 |
| Müller et al. (2022)b | Germany | inpatients | clinical | 34 | 64 | PHQ-9 |  | 0,51 | 861 |
| Natoli et al. (2016) | USA | undergraduates | non-clinical | 21,6 | 75,4 | BDI-II |  | 0,56 | 414 |
| Nordahl et al. (2024)b | Norway | community volunteers with propable GAD | non-clinical | 34,29 | 79,3 | PHQ-9 |  | 0,53 | 135 |
| Nordahl et al. (2021)b | Norway | undergraduates | non-clinical | 22,35 | 77,7 | HSCL-25 depression |  | 0,469 | 296 |
| Rakhimov et al. (2023)a | UK | community volunteers | non-clinical | 39 | 59,4 | PROMIS-SF depression |  | 0,52 | 468 |
| Ringwald et al. (2024)a | USA | community volunteers | non-clinical | 28 | 51,1 | CES-D |  | 0,213 | 311 |
| Salerno et al. (2015) | Italy | treatment-seeking obese adults | clinical | 45,19 | 79,86 | POMS-D |  | 0,44 | 422 |
| Schmitz et al. (2000)b | Germany | outpatients | clinical | 37 | 60,4 | SCL-90R-Depression |  | 0,61 | 1886 |
| Shin & Newman (2019)b | USA | undergraduates | non-clinical | 18,72 | 78 | BDI-II |  | 0,3075 | 369 |
| Sun et al. (2017) | China | undergraduates | non-clinical | 20,85 | 49,8 | SDS |  | 0,51 | 257 |
| Vierl et al. (2023) | Germany | treatment-seeking outpatients | clinical | 34,5 | 70,7 | PHQ-9 |  | 0,52 | 341 |
| Wei et al. (2003) | USA | undergraduates | non-clinical | 18,93 | 68 | BDI |  | 0,37 | 515 |
| Wei et al. (2021)a | USA | undergraduates | non-clinical | 19,26 | 68 | DASS-Depression |  | 0,3075 | 423 |
| Wei et al. (2005)a | USA | undergraduates | non-clinical | 19,88 | 65 | DASS-Depression |  | 0,39 | 229 |
| Wiseman et al. (2007)a | Israel | community volunteers | non-clinical | 26,23 | 48,23 | MHI-Depression |  | 0,45 | 141 |
| Zimmermann et al. (2013) | Austria | inpatients (and 19 healthy controls) | clinical | 32,8 | 87,3 | BDI |  | 0,4625 | 118 |

*Note. k* = 39, BSI = Brief Symptom Inventory (depression subscale), BDI = Beck Depression Inventory, GDS = Geriatric Depression Scale, SCL = Symptom Checklist (depression subscale), CES-D = Center for Epidemiologic Studies Scale (depression subscale), PHQ = Patient Health Questionnaire, DASS = Depression Anxiety Stress Scale (depression subscale), HSCL = Hopkins Symptom Checklist (depression subscale), PROMIS-SF = Patient-Reported Outcomes Measurement Information System (short-form, depression subscale), POMS-D = Profile of Mood States (depression subscale), SDS = Self-Rating Depression Scale, MHI = Mental Health Index (depression subscale)

**S2.2 Forest Plot**


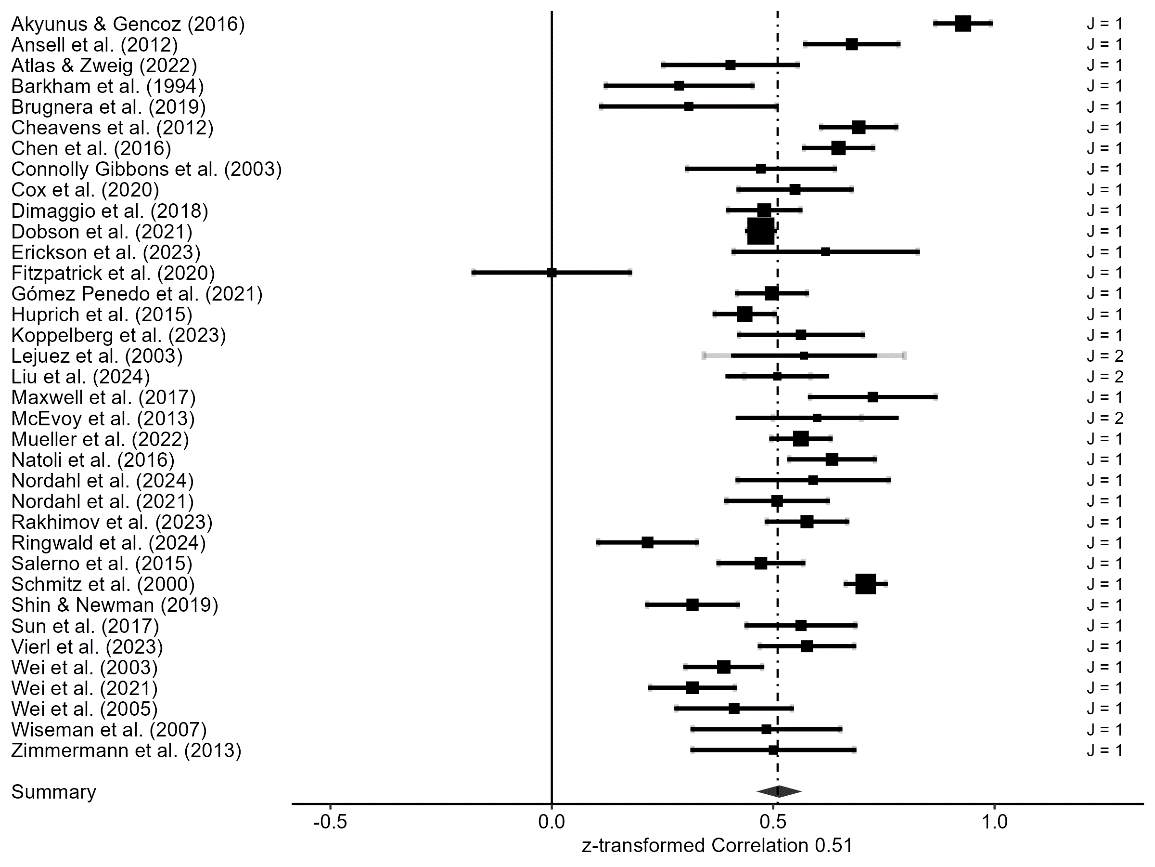


**S2.3 Funnel Plot**


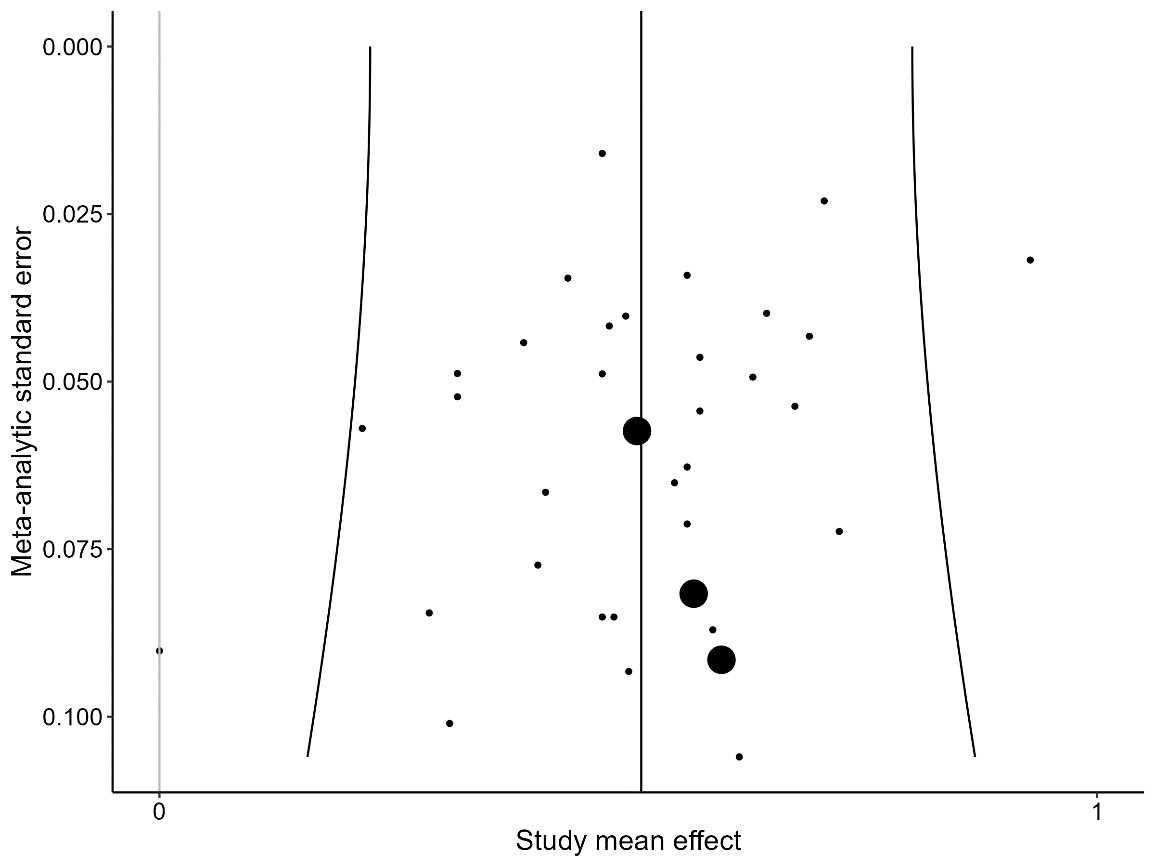


**S3 Interpersonal Problems and Symptoms of Anxiety**

**S3.1 Table** Study and Effect Size Characteristics for Studies Assessing Symptoms of Anxiety

|  | Study characteristic | | | | | |  | Effect size  characteristics | |
| --- | --- | --- | --- | --- | --- | --- | --- | --- | --- |
| Publication | Country | Population | Clinical  status | Mean age | Female  % | Mental health measure |  | Correlation coefficient (*r*) | Sample size |
| Akyunus & Gencoz (2016)c | Turkey | community volunteers | non-clinical | 26,85 | 68,34 | BSI-Anxiety |  | 0,49 | 988 |
| Barkham et al. (1994)c | UK | seeking psychotherapy | clinical | 38,96 | 49,1 | SCL-90R-Anxiety |  | 0,37 | 143 |
| Benecke et al. (2008)b | Austria | mixed patient sample | clinical | 34,5 | 83,3 | EER |  | 0,43 | 91 |
| Chen et al. (2016)b | China | undergraduates (possible mobile phone addicts) | non-clinical | 20,12 | 57,41 | SCS |  | 0,41 | 634 |
| Connolly Gibbons et al. (2003)b | USA | patients | clinical | 37,27 | 49 | BAI |  | 0,33 | 141 |
| Dally et al. (2005)a | Germany | patients with social anxiety | clinical | 33,1 | 69,5 | SIAS |  | 0,69 | 422 |
| Dally et al. (2005)b | Germany | patients with social anxiety | clinical | 33,1 | 69,5 | SPS |  | 0,629 | 422 |
| Dimaggio et al. (2018)c | Italy | treatment-seeking outpatients | clinical | 35,8 | 54,3 | STAI-Y State |  | 0,273 | 578 |
| Dobson et al. (2021)a | Canada | community volunteers | non-clinical | 44,13 | - | GAD-7 |  | 0,43 | 3932 |
| Erickson et al. (2023)a | USA | undergraduates | non-clinical | 19,56 | 76 | GAD-Q-IV |  | 0,52 | 92 |
| Juen et al. (2024) | Germany | treatment-seeking outpatients | clinical | 35,02 | 71 | PHQ-GAD |  | 0,36 | 421 |
| Kim & Bae (2022) | Republic of Korea | undergraduates | non-clinical | 22,42 | 55,2 | SIAS |  | 0,813 | 377 |
| Koppelberg et al. (2023)a | Germany | community volunteers | non-clinical | 23,97 | 50 | STAI State |  | 0,35 | 200 |
| Liu et al. (2024)b | China | undergraduates | non-clinical | 20,26 | 59,5 | GAD-7 |  | 0,45 | 511 |
| Liu et al. (2024)f | China | male offenders | non-clinical | 40,87 | 0 | GAD-7 |  | 0,49 | 979 |
| Malivoire & Koerner (2022) | Canada | community volunteers | non-clinical | 31,7 | 59,3 | GAD-Q-IV |  | 0,27 | 59 |
| McEvoy et al. (2013)b | Australia | anxiety and depression patients | clinical | 36,42 | 65 | BAI |  | 0,27 | 450 |
| McEvoy et al. (2013)e | Australia | eating disorder patients | clinical | 25,65 | 98,2 | DASS-A |  | 0,59 | 334 |
| Nordahl et al. (2024)a | Norway | community volunteers with propable GAD | non-clinical | 34,29 | 79,3 | BAI |  | 0,55 | 135 |
| Nordahl et al. (2021)a | Norway | undergraduates | non-clinical | 22,35 | 77,7 | HSCL-25 anxiety |  | 0,465 | 296 |
| Rakhimov et al. (2023)b | UK | community volunteers | non-clinical | 39 | 59,4 | PROMIS-SF anxiety |  | 0,47 | 468 |
| Schmitz et al. (2000)c | Germany | outpatients | clinical | 37 | 60,4 | SCL-90R-Anxiety |  | 0,45 | 1886 |
| Shin & Newman (2019)a | USA | undergraduates | non-clinical | 18,72 | 78 | GAD-Q-IV |  | 0,29875 | 369 |
| Wei et al. (2021)b | USA | undergraduates | non-clinical | 19,26 | 68 | DASS-Anxiety |  | 0,325 | 423 |
| Wei et al. (2005)b | USA | undergraduates | non-clinical | 19,88 | 65 | DASS-Anxiety |  | 0,31 | 229 |
| Wiseman et al. (2007)b | Israel | community volunteers | non-clinical | 26,23 | 48,23 | MHI-Anxiety |  | 0,5 | 141 |

*Note*. *k* = 26; BSI = Brief Symptom Inventory (anxiety subscale), SCL = Symptom Checklist (anxiety subscale), EER = [Fragebogen zur Erfassung von Emotionserleben und Emotionsregulation] (Questionnaire for Assessing Emotional Experience and Emotion Regulation, anxiety subscale), SCS = Self-Consciousness Scale (social anxiety subscale), BAI = Beck Anxiety Inventory, SIAS = Social Interaction Anxiety Scale, SPS = Social Phobia Scale, STAI = State-Trait Anxiety Inventory, GAD-7 = Generalized Anxiety Disorder, GAD-Q-IV = Generalized Anxiety Disorder Questionnaire IV, DASS = Depression Anxiety Stress Scale (anxiety subscale), HSCL = Hopkins Symptom Checklist (anxiety subscale), PROMIS-SF = Patient-Reported Outcomes Measurement Information System (short-form, anxiety subscale), MHI = Mental Health Index (anxiety subscale).

**S3.2 Forest Plot**


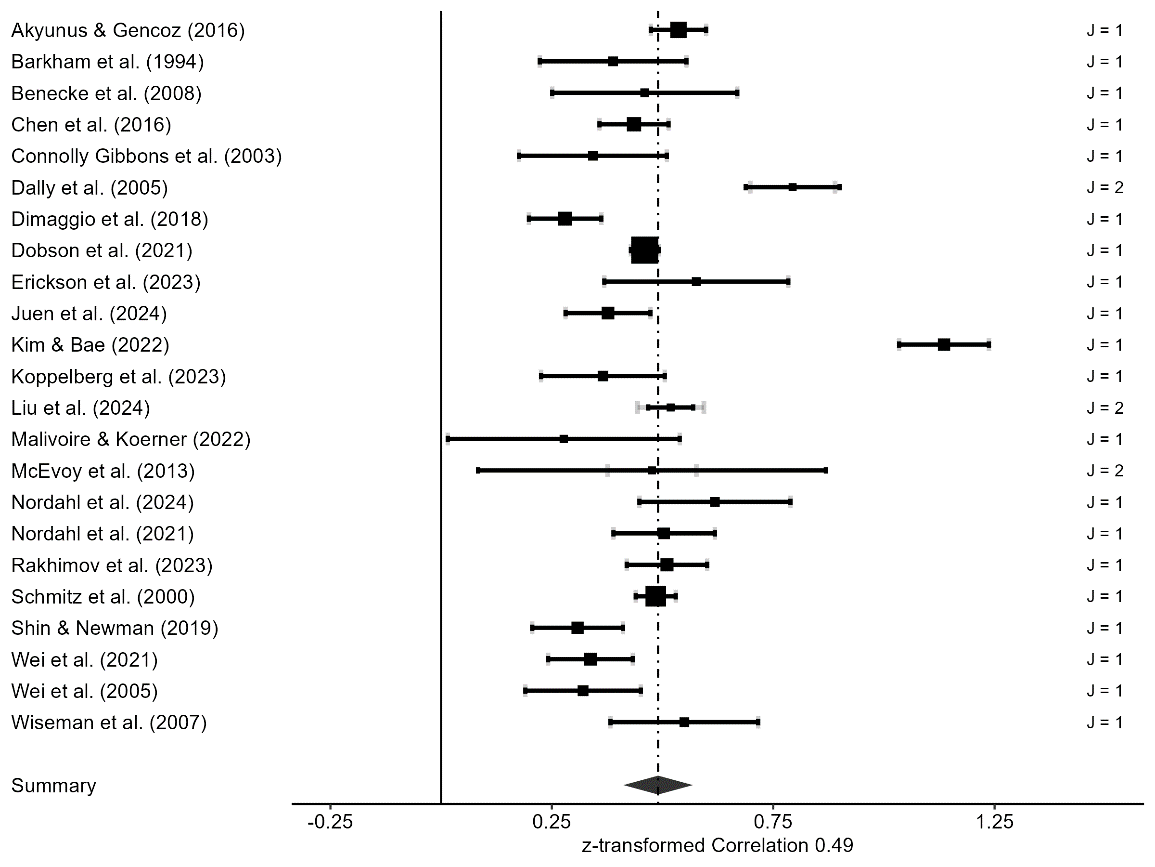


**S3.3 Funnel Plot**


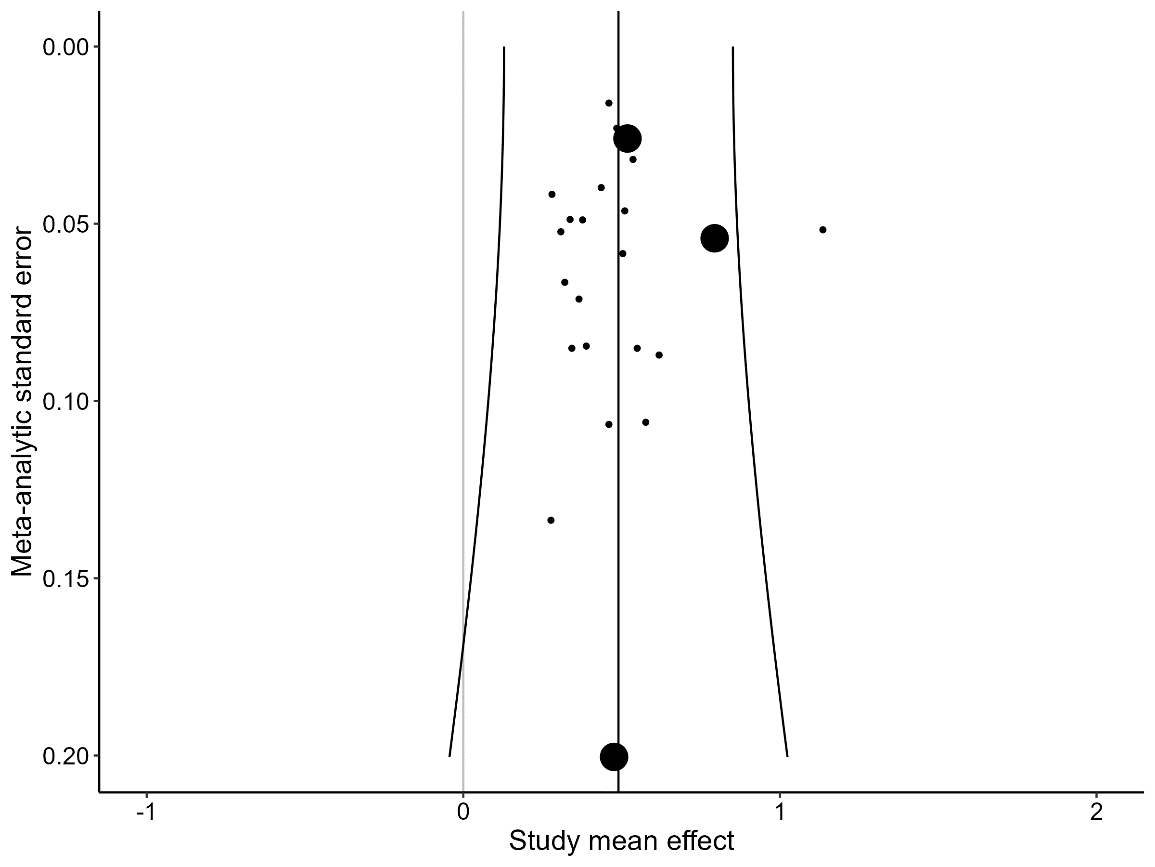


**S4 Interpersonal Problems and Positive Emotions**

**S4.1 Table** Study and Effect Size Characteristics for Studies Assessing Positive Emotions

|  | Study characteristic | | | | | |  | Effect size  characteristics | |
| --- | --- | --- | --- | --- | --- | --- | --- | --- | --- |
| Publication | Country | Population | Clinical  status | Mean age | Female  % | Mental health measure |  | Correlation coefficient (*r*) | Sample size |
| Akyunus & Gencoz (2016)d | Turkey | community volunteers | non-clinical | 26,85 | 68,34 | PANAS |  | -0,22 | 1002 |
| Benecke et al. (2008)b | Austria | mixed patient sample | clinical | 34,5 | 83,3 | EER |  | -0,32 | 92 |
| Gurtman (1992b)a | USA | undergraduates | non-clinical | - | 53,37 | PANAS |  | -0,216 | 163 |
| Liu et al. (2024)c | China | undergraduates | non-clinical | 20,26 | 59,5 | PANAS |  | -0,12 | 511 |
| Liu et al. (2024)g | China | male offenders | non-clinical | 40,87 | 0 | PANAS |  | -0,03 | 555 |
| Nicolaou et al. (2023) | USA | community volunteers | non-clinical | 35,37 | 39,08 | PANAS |  | -0,514 | 238 |
| Prout et al. (2021)b | USA | treatment-seeking outpatients | clinical | 29,88 | 75,4 | PANAS |  | -0,22 | 142 |
| Ringwald et al. (2024)c | USA | community volunteers | non-clinical | 28 | 51,1 | PANAS |  | -0,262 | 311 |
| Tracey et al. (1996)b | USA | undergraduates | non-clinical | - | 58,1 | PANAS |  | 0,09 | 105 |

*Note. k* = 9, PANAS = Positive and Negative Affect Schedule (positive emotions subscale), EER = EER = [Fragebogen zur Erfassung von Emotionserleben und Emotionsregulation] (Questionnaire for Assessing Emotional Experience and Emotion Regulation, joy subscale)

**S4.2 Forest Plot**


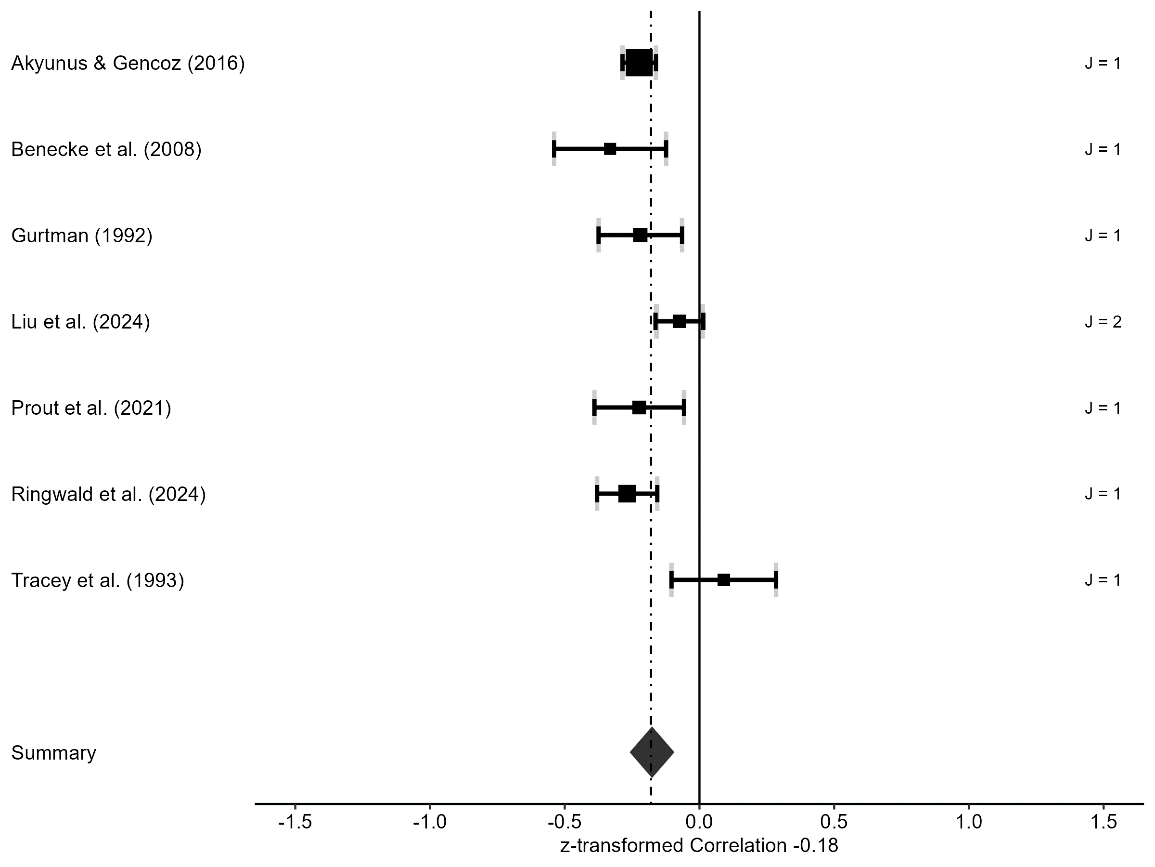


**S4.3 Funnel Plot**


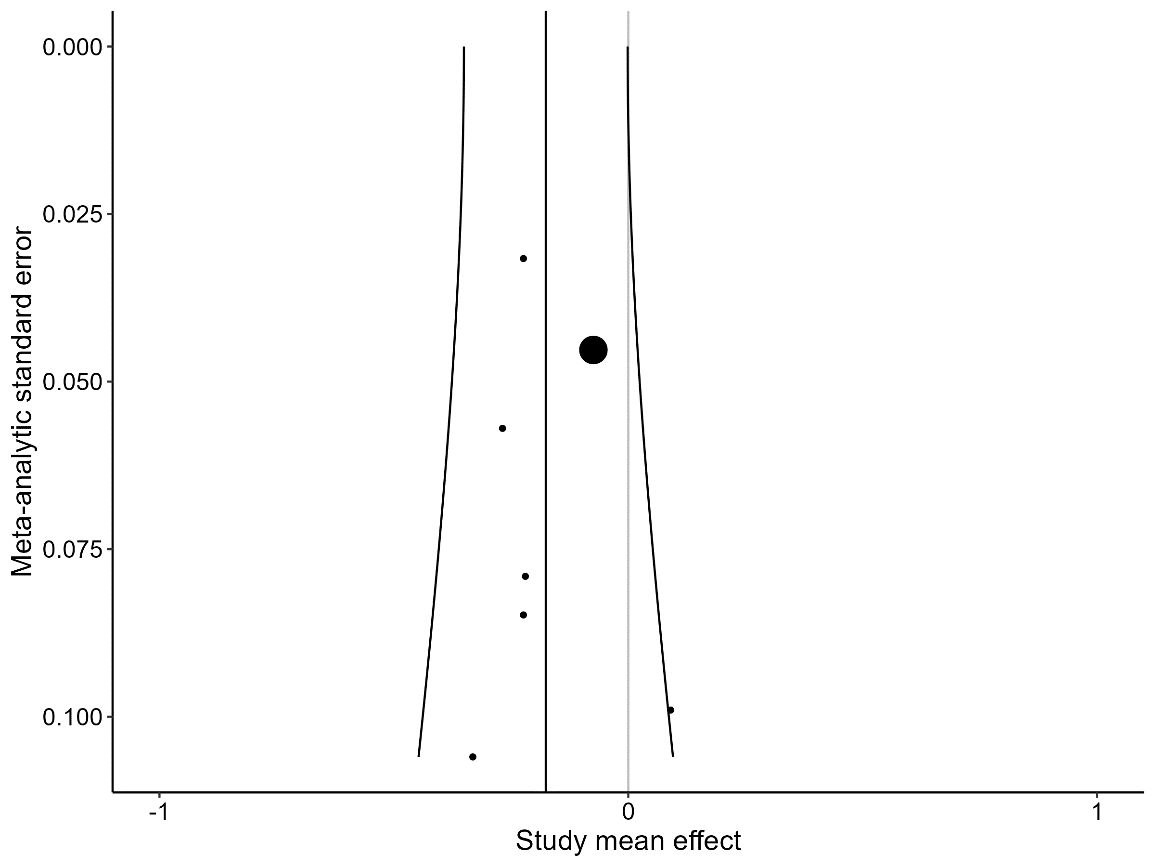


**S5 Interpersonal Problems and Negative Emotions**

**S5.1 Table** Study and Effect Size Characteristics for Studies Assessing Negative Emotions

|  | Study characteristic | | | | | |  | Effect size  characteristics | |
| --- | --- | --- | --- | --- | --- | --- | --- | --- | --- |
| Publication | Country | Population | Clinical  status | Mean age | Female  % | Mental health measure |  | Correlation coefficient (*r*) | Sample size |
| Akyunus & Gencoz (2016)e | Turkey | community volunteers | non-clinical | 26,85 | 68,34 | PANAS |  | 0,45 | 1002 |
| Gurtman (1992b)b | USA | undergraduates | non-clinical | - | 53,37 | PANAS |  | 0,637 | 163 |
| Liu et al. (2024)d | China | undergraduates | non-clinical | 20,26 | 59,5 | PANAS |  | 0,45 | 511 |
| Liu et al. (2024)h | China | male offenders | non-clinical | 40,87 | 0 | PANAS |  | 0,46 | 555 |
| Prout et al. (2021)c | USA | treatment-seeking outpatients | clinical | 29,88 | 75,4 | PANAS |  | 0,48 | 142 |
| Ringwald et al. (2024)d | USA | community volunteers | non-clinical | 28 | 51,1 | PANAS |  | 0,483 | 311 |
| Tracey et al. (1996)c | USA | undergraduates | non-clinical | - | 58,1 | PANAS |  | 0,46 | 105 |

*Note. k* = 7, PANAS = Positive and Negative Affect Schedule (negative emotions subscale)

**S5.2 Forest Plot**


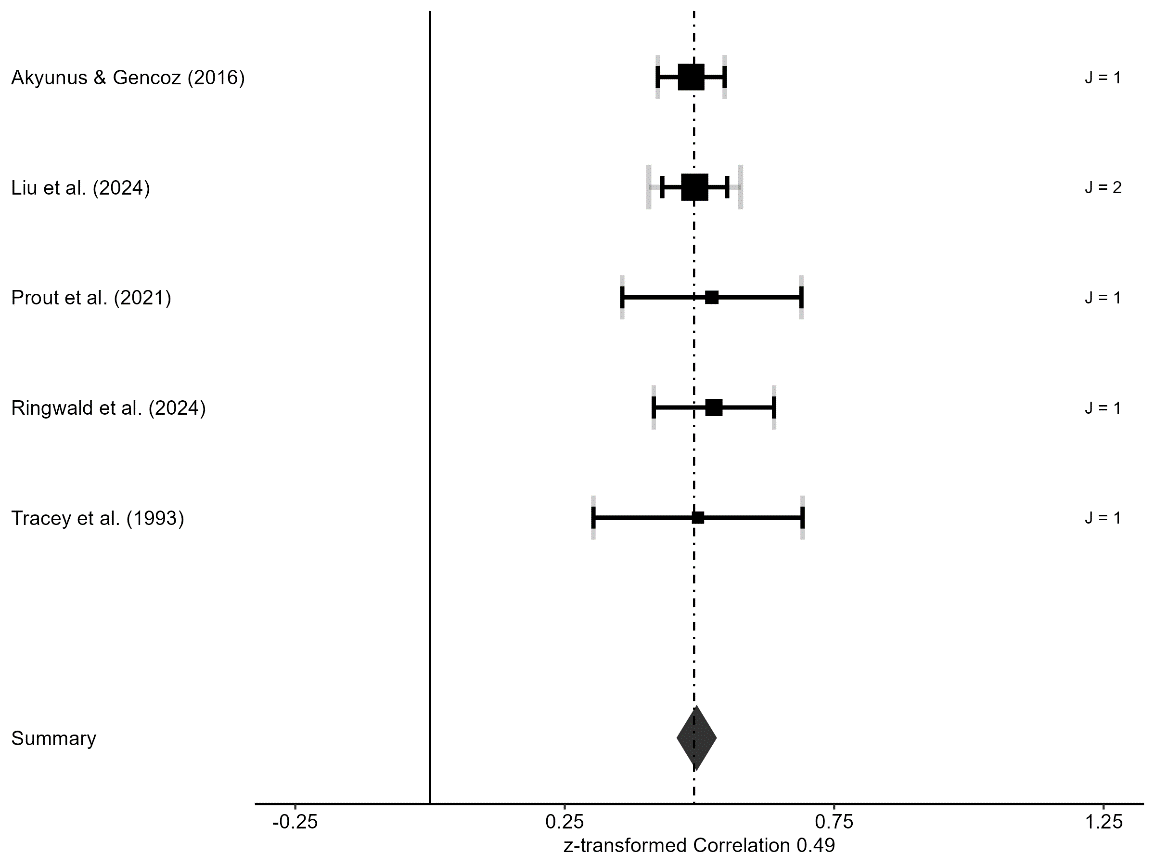


**S5.3 Funnel Plot**


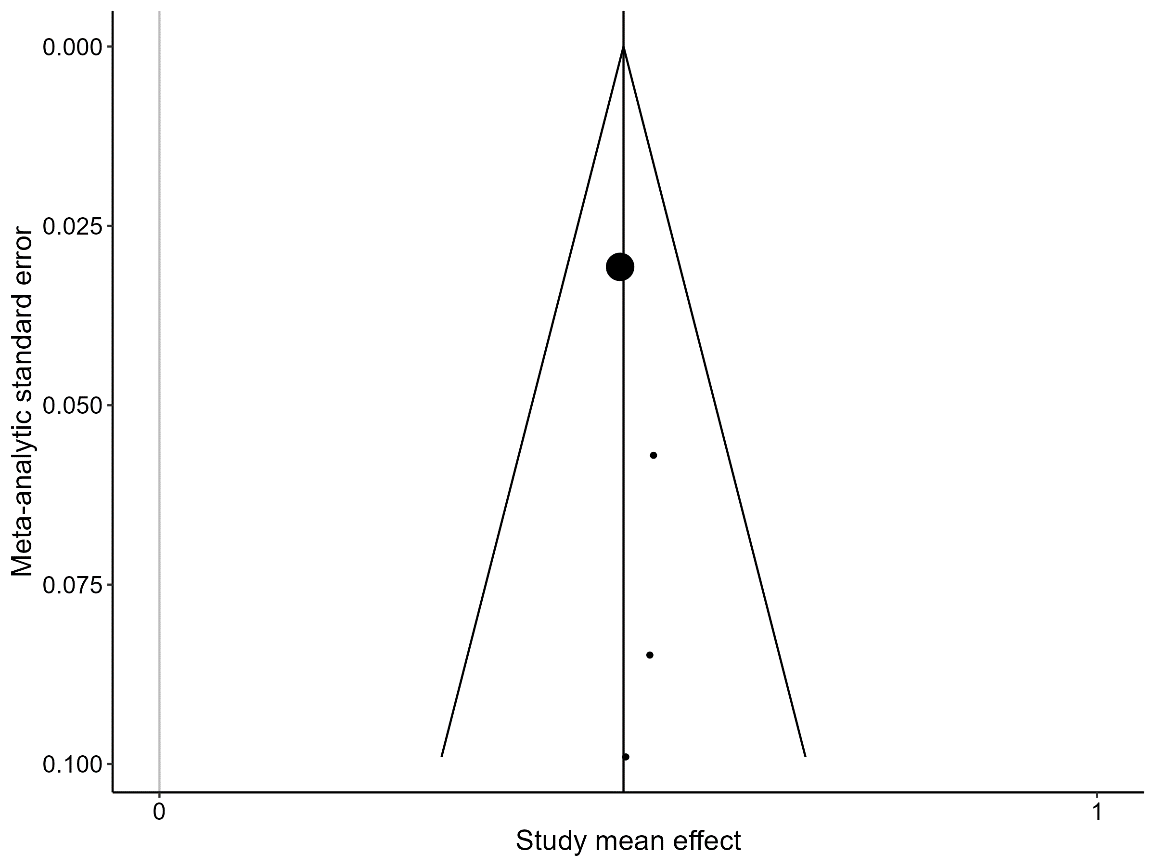


**S6 Interpersonal Problems and Well-Being**

**S6.1 Table** Study and Effect Size Characteristics for Studies Assessing Well-Being

|  | Study characteristic | | | | | |  | Effect size  characteristics | |
| --- | --- | --- | --- | --- | --- | --- | --- | --- | --- |
| Publication | Country | Population | Clinical  status | Mean age | Female  % | Mental health measure |  | Correlation coefficient (*r*) | Sample size |
| González-Cifuentes & Ruiz (2022) | Colombia | community volunteers | non-clinical | 34 | 55,2 | SWLS |  | -0,317 | 701 |
| Liu et al. (2024)i | China | male offenders | non-clinical | 40,87 | 0 | SHS |  | -0,26 | 424 |
| McEvoy et al. (2013)c | Australia | anxiety and depression patients | clinical | 36,42 | 65 | Q-LES-Q |  | -0,42 | 486 |
| McEvoy et al. (2013)f | Australia | eating disorder patients | clinical | 25,65 | 98,2 | Q-LES-Q |  | -0,54 | 327 |
| Müller et al. (2022)c | Germany | inpatients | clinical | 34 | 64 | WHO-5 |  | -0,35 | 861 |
| Rakhimov et al. (2023)c | UK | community volunteers | non-clinical | 39 | 59,4 | WEMWBS |  | -0,52 | 468 |
| Wei et al. (2021)c | USA | undergraduates | non-clinical | 19,26 | 68 | BMSLSS |  | -0,24 | 423 |

*Note. k* = 7; SWLS = Satisfaction with Life Scale, SHS = Subjective Happiness Scale, Q-LES-Q = Quality of Life Enjoyment and Satisfaction Questionnaire, WHO-5 = WHO-5 Well-Being Index, WEMWBS = Warwick-Edinburgh Mental Well-Being Scale, BMSLSS = Brief Multidimensional Students’ Life Satisfaction Scale.

**S6.2 Forest Plot**

**S6.3 Funnel Plot**
